# Polarized Trout Epithelial Cells Regulate Transepithelial Electrical Resistance, Gene Expression, and the Phosphoproteome in Response to Viral Infection

**DOI:** 10.3389/fimmu.2020.01809

**Published:** 2020-08-14

**Authors:** Shankar C. Mandal, Manfred Weidmann, Amaya Albalat, Emma Carrick, Bernat Morro, Simon MacKenzie

**Affiliations:** ^1^Institute of Aquaculture, University of Stirling, Stirling, United Kingdom; ^2^Department of Fisheries, University of Dhaka, Dhaka, Bangladesh; ^3^Institute of Cardiovascular and Medical Sciences, University of Glasgow, Glasgow, United Kingdom

**Keywords:** RTgill-W1 cell, transepithelial electrical resistance, poly(I:C), synthetic dsRNA, SAV-2, Rig-like receptor, protein phosphorylation, rainbow trout

## Abstract

The burden of disease is a major challenge in aquaculture production. The fish gill characterized with a large surface area and short route to the bloodstream is a major environmental interface and a significant portal of entry for pathogens. To investigate gill responses to viral infection the salmonid gill cell line RTgill-W1 was stimulated with synthetic dsRNA and the salmonid alphavirus subtype 2 (SAV-2). Epithelial integrity in polarized cells can be measured as transepithelial electrical resistance (TEER) which is defined as the electrical resistance across a cell monolayer. TEER is a widely accepted quantitative measure of cellular integrity of a cell monolayer. TEER increased immediately after stimulation with the synthetic dsRNA, polyinosinic:polycytidylic acid (poly(I:C)). In parallel, tight junction and gene expression of innate immune activation markers was modulated in response to poly(I:C). The SAV-2 virus was found to replicate at a low level in RTgill-W1 cells where TEER was disturbed at an early stage of infection, however, gene expression related to tight junction regulation was not modulated. A strong poly(I:C)-driven antiviral response was observed including increases of Rig-like receptors (RLRs) and interferon stimulating genes (ISGs) mRNAs. At the level of signal transduction, poly(I:C) stimulation was accompanied by the phosphorylation of 671 proteins, of which 390 were activated solely in response to the presence of poly(I:C). According to motif analysis, kinases in this group included MAPKs, Ca2+/calmodulin-dependent kinase (CaMK) and cAMP-dependent protein kinase (PKA), all reported to be activated in response to viral infection in mammals. Results also highlighted an activation of the cytoskeletal organization that could be mediated by members of the integrin family. While further work is needed to validate these results, our data indicate that salmonid gill epithelia has the ability to mount a significant response to viral infection which might be important in disease progression. *In vitro* cell culture can facilitate both a deeper understanding of the anti-viral response in fish and open novel therapeutic avenues for fish health management in aquaculture.

## Introduction

In fish, the gills consist of four pairs of vascularised gill arches composed of hundreds of gill filaments, which increase contact surface by folding into the secondary lamella (1). Gills are in direct contact with the water and therefore are continuously exposed to environmental insults. Thus, the gills act as an important organ of entry for pathogens including intracellular bacteria and viruses (2). There is an evident need for the fish to defend and protect such a large and delicate surface from pathogen invasion (1). The fish gill epithelium covering the gill filaments and lamellae separates the external environment from extracellular fluids thus plays a critical role in gill function. Epithelial cells form intercellular junctions which result in a tight cellular barrier these tight junctions control the diffusion of different molecules across the paracellular compartments. In fish tight junctions are found in gill epithelium as well as intestine, skin, muscle, brain, blood-brain barrier, vascular system, swim bladder, lateral line, gall bladder, kidney, head kidney and spleen in different teleost fishes [(3); reviewed in (4)].

Tight junctions are important components of the epithelial junctional complex and form a circumferential, belt-like structure at the luminal end of the intercellular space acting as a gatekeeper of the paracellular pathway (5). Transepithelial electrical resistance (TEER) is the electrical resistance between the apical and basal side of the epithelial (or endothelial) cells. In *in vitro* studies the TEER of an epithelial monolayer can be measured in transwell systems where there is a positive correlation between the development of a tight junction between adjacent cells and TEER. The TEER measurement has been widely used and is a reliable, convenient and non-destructive method. The TEER value is a strong indicator of cell integrity where higher values of TEER indicate increased cellular integrity. This quantitative expression of barrier integrity is expressed as ohms-cm^2^ (Ω-cm^2^) (6). The complexity of the tight junction network, has an effect on TEER and Claude and Goodenough (7) demonstrated a direct relationship between TEER and the number of parallel strands between cells.

In gills, innate and adaptive immune response related molecules including cytokines, caspases, immunoglobulins and major histocompatibility complex (MHC) have been described (8, 9). More recently, Boison et al. (10) have reported the modification of thousands of genes by transcriptome analysis in salmon gills in response to amoebic gill disease (AGD). Furthermore, gill mucosal immunoglobulins have been shown to recognize gill microbiota in rainbow trout, *Oncorhynchus mykiss* (11) although no changes were observed in gill microbiome profiles in *Flavobacterium psychrophilum* resistant and susceptible lines of rainbow trout (12). These studies highlight a role for the gill in the fish immune response and the complexity of host-pathogen interactions at the epithelial cell surface. The use of cell cultures such as the RTgill-W1 cell line developed from a primary culture of rainbow trout gill *Oncorhynchus mykiss* (Walbaum) (13) can be very useful to delineate specific molecular and cellular activation pathways. The RTgill-W1 cell line has been used to study the antiviral response (14), the effect of osmoregulatory hormones (15) and in ecotoxicological studies (16). In RTgill-W1 cells cultured under transwell conditions canonical features of normal epithelial function are observed such as pavement cells (PVCs) and microridges however are absent in flask culture. Cells cultured on transwells also have the transport properties which are absent in flask cultures (16).

The host response against viral and bacterial infections relies firstly on the recognition of pathogens by a number of host receptors such as the pathogen recognition receptor family (PRR). Antiviral immunity is activated mostly by cytosolic PRRs including the Toll-Like Receptor 3 (TLR3) and the RIG- like Receptors (RLRs) including MDA5 (melanoma differentiation-associated gene 5), RIG-I (retinoic acid-inducible gene I) and LGP2 (laboratory of genetics and physiology 2). RLRs are cytosolic pattern recognition receptors and are broadly expressed in most tissues including epithelial cells (17) where they signal innate immune activation. RIG-I and MDA5 detect a variety of viruses that trigger signal downstream to initiate the production of IFN and induction of an antiviral response while LGP2 regulates MDA5 and RIG-I signaling (18).

During their replication cycles most RNA viruses produce double stranded RNA (dsRNA), which acts as a strong type I IFN inducer. Both natural and synthetic dsRNAs are known to induce type I interferons and the production of other cytokines. Poly(I:C), a structural analog of dsRNA, binds to toll-like receptor 3 (TLR3) and it has been widely used as an immuno-stimulant in humans and mice against viral diseases based on its ability to enhance innate and adaptive immunity (19). As observed in mammals, dsRNA-driven activation of the antiviral response *in vivo* (20) and *in vitro* in Teleost fish (21, 22) has been reported. In fish, TLR22 has been shown to be induced by poly(I:C) in common carp (23) and to recognize poly(I:C) in fugu with subsequent induction of interferon resulting in protection from birnavirus infection (24). Apart from TLR3 and TLR22, both TLR7 and TLR8 have been identified as PRRs with potential to respond to dsRNA in different fish species including rainbow trout although in trout their expression was not poly(I:C)-inducible (25). However, in large yellow croaker, *Larimichthys crocea*, poly(I:C)-stimulation has been shown to induce both TLRs in different organs including the gill (26).

Viral pathogens are the predominant agents causing a substantial loss to aquaculture production. Salmonid alphavirus (SAV) causes pancreas disease (PD) and sleeping disease (SD) in farmed Atlantic salmon (*Salmo salar*) and rainbow trout (*Oncorhynchus mykiss*) in Europe (27). Recent molecular taxonomic studies describe six salmonid alphavirus subtypes all of which causing PD in salmon. SAV-2 has also been reported to causes SD in freshwater rainbow trout in several European countries (28). Fish RNA viruses and the pathology they cause are relatively well-characterized, however, the understanding of how these viruses and in particular SAV manipulates the host's machinery during their replication cycle is extremely limited. SAV-2 is however a +ssRNA virus that as such should be detected by TLR3 and RLR initiating an antiviral response in fish.

In recent studies, two newly developed Atlantic salmon gill cell lines have been shown to be permissive to some salmonid viruses including infectious pancreatic necrosis virus (IPNV), infectious hematopoietic necrosis virus (IHNV) and viral haemorrhagic septicaemia virus (VHSV) (29). Although, the mechanism by which dsRNA is recognized by the fish gill epithelia has not yet been elucidated. In the present study, cellular response of the salmonid epithelial cell line, RTgill-W1, to dsRNA challenge was characterized. The response of RTgill-W1 cells to SAV-2 at cellular level and molecular level was also studied. We used a combination of transepithelial electrical resistance (TEER) and tight junction gene expression to examine epithelial integrity and barrier status. Additionally, we used gene expression analysis and phosphoproteomics to study signal transduction of critical responses. Our results highlight an integrated response to viral infection upon PAMP challenge in fish epithelia that is conserved across the vertebrates.

## Materials and Methods

### Cellular and Molecular Responses of RTgill-W1 Cells Upon Poly(I:C) Stimulation and SAV-2 Infection

#### Gill Cell Line

The RTgill-W1 cell line derived from rainbow trout gill (13) was used in the current study. Cells were maintained in Leibowitz L-15 media supplemented with L-glutamax (GIBCO Life Technologies) and 10% of fetal bovine serum (FBS) (Life Technology) at 22 °C in 75 cm^2^ plastic flasks (SARSTEDT, Germany). Cells were sub-cultured once a week by trypsinising with 0.05% trypsin–EDTA (GIBCO Life Technologies). Experiments were conducted using 12 or 6 well transwells (BD Falcon) with pore size of 0.4 μm and a pore density of 1.6 × 10^6^ pores per cm^2^.

#### Virus Culture and Titration

Three fish cell lines CHSE-214, CHH-1 and TO were initially tested for growing SAV-2 (Isolate V0702, Passage 1, cell line: CHH-1). Serial dilutions of the virus stock were inoculated by adsorption inoculation and simultaneous inoculation. CHSE-214 cells inoculated by adsorption with a virus stock dilution of 10^−3^ were found to facilitate best growth of SAV-2. Then SAV-2 was bulked up by inoculating CHSE-214 cell line in 25 cm^2^ TC flasks with the same stock dilution as above. Virus was harvested at day 20 of inoculation and kept at −70°C until further use. Virus titer was carried out in CHSE-214 cells and a TCID_50_ of 10^6.35±0.2^ /ml was determined.

#### Measurement of Trans-Epithelial Electrical Resistance (TEER)

RTgill-W1 cells were seeded onto 12 well transwells with a growth area of 0.9 cm^2^. The apical compartment of the transwells contained RTgill-W1 cells in L-15 medium supplemented with 10% FBS and L-Glutamax while the basolateral compartment contained only complete growth medium. TEER measurements were performed with a chopstick-type probe (STX-2) connected to a Millicell ERS-2 voltmeter (EMD Millipore Corporation, Billerica, USA), according to the manufacturer's instructions. The resistance measured across a culture insert with no seeded cells was used for background correction of all TEER measurements. Post seeding TEER was measured at different time points (0, 3, 6, 24, 48, and 72 h) until TEER values reached stability. When TEER values stabilized, cells from the treatment group were stimulated with Poly(I:C) (InvivoGen, San Diego, USA) at a concentration of 10 μg/ml. During stimulation, medium from the apical compartments of control and blank groups was replaced with fresh medium. Three independent experiments each in triplicate were conducted and TEER was measured in each well at each time point three times. Post-stimulation TEER was measured at 0, 3, 6, 24, 48 and 72 h. TEER value was calculated as:

$$\begin{array}{l} Unit\;Area\;Resistance\;\left(\Omega {-cm}^{2}\right)\\ \;=Resistance\;\left(\Omega \right)\times Effective\;membrane\;Area\;\left(cm^{2}\right). \end{array}$$

The unit area resistance was obtained by multiplying the meter reading by the effective membrane area of the insert.

To investigate the effects of SAV-2 on the cellular integrity RTgill-W1, cells were infected with SAV-2 at the multiplicity of infection (MOI) 10, 1.0, and 0.1 and TEER was measured 0.5, 1, 3, 6, 24, 48, 72, and 96 h post infection.

#### Quantitative Real Time PCR (qPCR)

For absolute quantification, a DNA standard for each innate immune response and tight junction gene target (Table 1) was generated. The PCR products were ligated into pGEM-T easy vector system (Promega) and transformed into *E*. *coli* DH5α competent cells (Thermo Fisher Scientific). Clones were cultured and selected on LB agar plates containing 100 μg/ml ampicillin. Plasmids were purified using the NucleoSpin plasmid DNA purification kit (Macherey-Nagel). Plasmids were subjected to LIGHTrun sequencing (GATC) to ensure that they contained the correct sequences. The copy number per microliter of plasmid DNA was calculated and, finally, the plasmid DNA was diluted in nuclease free water in a range from 1 × 10^7^ copy/μl to 1 × 10^1^ copy/μl and used as standard for absolute quantification of mRNA expression in the samples.

**Table 1:** Primer sequences used for RT-PCR and RT-qPCR of rainbow trout transcript targets with Co-efficient of determination (*R*^2^), efficiency, and sensitivity for the target genes generated from the standard curve.

**Name of the gene**	**Sequence (5′-3′)**	**Ann. Temp.(°C)**	**Amplicon size (bp)**	**Reference sequence Acc. No**.	**R^**2**^**	**Efficiency (E)**	**N**	**Sensitivity**	**Application**
T-Claudin-3a	F-TGGATCATTGCCATCGTGTC R- GCCTCGTCCTCAATACAGTTGG	60	139	BK007964	0.96	1.72	3	10^1^	RT-PCR, RT-qPCR
T-Claudin-8d	F-GCAGTGTAAAGTGTACGACTCTCTG R- CACGAGGAACAGGCATCC	60	339	BK007966	0.98	1.71	3	10^2^	RT-PCR, RT-qPCR
T-ZO-1	F-AAGGAAGGTCTGGAGGAAGG R- CAGCTTGCCGTTGTAGAGG	59	291	HQ656020	0.99	1.93	3	10^1^	RT-PCR, RT-qPCR
T-TLR3	F-AGCCCTTTGCTGCCTTACAGAG R-GTCTTCAGGTCATTTTTGGACACG	60	61	CA363490	0.99	1.93	3	10^1^	RT-qPCR
T- rtIFN2	F-GACGTCTGTCACGTGGAACAAAAT R-CCAAACACCGCCCACAACA	59	100	NP_001153974	0.97	2.24	3	10^1^	RT-PCR, RT-qPCR
T-Mx2	F-GATGCTGCACCTCAAGTCCT R-TAGCTGCGTGCCTTCATCAG	60	237	RBTMx2/RBTMx3	0.99	1.95	3	10^1^	RT-qPCR
S-RIG-I	F-ACTGATCGGGAGAGGACACAA R-CTTGACCACATTGCCAACGTAT	59	202	XM_021593781	0.99	1.88	3	10^2^	RT-PCR, RT-qPCR
T-MDA5	F-AGAGCCCGTCCAAAGTGAAGT R-GTTCAGCATAGTCAAAGGCAGGTA	59	357	NM_001195179	0.99	1.84	3	10^1^	RT-PCR, RT-qPCR
T-LGP2b	F-GTGGCAGGCAATGGGGAATG R-CCTCCAGTGTAATAGCGTATCAATCC	59	212	FN396358	0.99	1.93	3	10^1^	RT-PCR, RT-qPCR
T-IPS1	F-AGCCAGCCATACTCAGGAGA R-CGTCCTCAGACACGTGAACA	59	268	NM_001195181	0.99	1.87	3	10^1^	RT-PCR, RT-qPCR
S-TBK1	F-GACCTGTATGCGGTGAAGGT R-CAGACTCCCACAGGGACAAT	59	161	XM_021592888	0.99	1.95	3	10^1^	RT-PCR, RT-qPCR
T-IRF3	F-TGTATACACAGCGGAGGGGA R-CACCCACAGCATCCTCCATT	59	209	NM_001257262	0.99	1.82	3	10^1^	RT-PCR, RT-qPCR
T-PKR	F-GGAAAGCTAAGCGGGAGGTT R-TCCTCTCGTCGATCCACACT	59	219	NM_001145891	0.99	1.80	3	10^1^	RT-PCR, RT-qPCR
S-ISG15	F-AAGTGATGGTGCTGATTACGG R-TTGGCTTTGAACTGGGTTACA	56	118	NM_001124609	0.98	2.01	3	10^2^	RT-PCR, RT-qPCR
T-VIG-1	F-CTCCAGCTCCCAAGTGTCAG R-TTGTACTTCCGGCACCAGTC	60	206	NM_001124253	0.99	1.95	3	10^2^	RT-PCR, RT-qPCR

*Efficiency was calculated using the formula, E =10^(−1/−slope)^. An E value of 2.0 is equivalent to 100% efficiency. N represents the number of qPCR repetitions for each gene to produce the standard values, while sensitivity is the lowest number of target molecule copies determined*.

SYBR Green based qPCR was performed to test the DNA standards using Luminaries Color HiGreen qPCR Master Mix (Thermo Scientific) and a Roche LightCycler 480 instrument using the following PCR temperature profile: 1 cycle of Pre-treatment at 50°C/2 min, initial denaturation at 95°C/10 min and 40 PCR cycles at 95°C/15s, 56–60°C (depending on primer sets)/30s, 72°C/30s (ramping rate 2.2°C/s), followed by dissociation curve: 95°C/10s (ramping rate 4.4°C/ s), 55°C/5s (ramping rate 2.2°C/ s), and 95°C/30s (ramping rate 4.4°C/ s), and cooling 40°C/30s (ramping rate 1.5°C/ s) to confirm the generation of a single specific amplicon. Two microlitres of diluted cDNA (1:5), SYBR Green (1x), and 400 nM of each primer in a total volume of 20 μl. Standard sets of each target were run in duplicate including no template controls. Three independent runs were conducted, and mean CT values were plotted against log of copy number to generate a standard curve. Efficiency (E) and coefficient of correlation (r^2^) of each target were determined (Table 1). For absolute quantification of each mRNA transcript, the copy number was extrapolated from the standard curve. The MIQE guideline (30) was followed in all the steps from RNA extraction to qPCR data analysis.

To generate the quantitative RNA standard for SAV-2 viral RNA was extracted from the virus supernatant using Roche High Pure Viral RNA, and reverse transcribed using the Transcriptor Onestep RT-PCR kit (Roche). Primers SD STDUP 5′-aagaaatgcaccaggttytccac-3′ and SD STD DP 5′-cacctctttgcctccgctg-3′ were used to amplify a 315 bp fragment of the E gene. The amplicon was purified using the DNA Clean & Concentrator (Zymo Research) and ligated into pCRII using the TA cloning kit (Invitrogen), and transformed into TOP 10 F' *E*. *coli* competent cells. Clones were incubated and selected at 37°C on LB agar plates supplemented with ampicillin (1 μg/ml). Plasmid DNA was extracted using the High Pure Plasmid Isolation Kit (Roche) and inserts confirmed by LIGHTrun sequencing (GATC). RNA transcription for a quantitative standard was performed as described by Weidmann et al. (31). TaqMan probe based real time RT-qPCR was performed to quantify the viral RNA copy number at defined time points and virus concentration using the LC 480 RNA Master Hydrolysis kit (Roche) the Roche LightCycler 2.0, the following primers and probe: SAVSDUP 5′-tccaccaccccgaagaagtc-3′, SAVSDDP 5′-atgtcaccacggtgctgatctc-3′ and probe SAVSDLNAP 5′- 6FAM-AATCGGCAGAGCGTC–BBQ-3′ (LNA nucleotides underscored), and using the following temperature profile: reverse transcription at 95°C/10 min (ramping rate 4.4°C/ s), initial denaturation at 95°C/10 min (ramping rate 4.4°C/ s), and 55 PCR cycles at 95°C/10s (ramping rate 4.4°C/ s), 58°C/20s (ramping rate 2.2°C), 72°C/15s (ramping rate 4.4°C/ s), and cooling 40°C/30s (ramping rate 1.5°C/ s).

#### RNA Extraction and cDNA Synthesis

Total RNA was extracted from cells grown onto 12-well transwells using TRI reagent (Sigma-Aldrich, St. Louis, MO, USA) according to the manufacturer's instructions with some modifications. RNA was quantified using a Nanodrop Spectrophotometer (ND-1000, Labtech International, Uckfield, UK). RNA integrity was assessed by agarose gel electrophoresis. For cytoplasmic RNA extraction, 0.1% NP-40 was added to 100 μl cell lysate (Thermo Fisher Scientific). The cytoplasmic fraction was collected by centrifuging for 1 min at 14,000 rpm at 4°C. RNA purity and quantity were determined by Nanodrop Spectrophotometer. RNA integrity was assessed by agarose gel electrophoresis. All extractions were repeated twice for reproducibility. Complementary DNA (cDNA) was synthesized from 500 ng of total RNA from each sample using Superscript III First-Stand Synthesis System (Invitrogen).

#### Gene Expression Analysis

To evaluate the effects of poly(I:C) stimulation on the expression of tight junction and immune genes in RTgill-W1, cells were grown to around 95% confluence in triplicate onto 12-well transwells. To equilibrate inserts to the medium, insert and wells were preloaded with 2 ml and 1 ml cell culture medium, respectively, in the basolateral and apical side. Each insert was seeded with 1.0 × 10^5^ RTgill-W1 cells, respectively. Cells were treated with or without poly(I:C) at 10 μg/ml for 6 and 24 h and then harvested for total RNA extraction. In the time course response experiment, cells in the transwells were stimulated with poly(I:C) at a concentration of 1 μg/ml for 0.5, 1, 3, 6, and 24 h. Afterwards, cells were harvested for total RNA extraction.

To investigate TLR3 mediated, RLR associated antiviral response, and tight junction response in RTgill-W1 cells upon SAV-2 infection, RTgill-W1 cells were grown onto 12-well transwells and cells were allowed to grow until 95% confluency. Cells were then infected with SAV-2 at MOI 10 and incubated for 6, 12, 18, 24, and 30 h. Uninfected control groups for each time point were maintained. Each treatment in both infected and uninfected groups was conducted in triplicate and cells from each treatment group were pooled for cytoplasmic and total RNA extraction.

In all cases each treatment was run in triplicate and the experiment was performed three times.

Extracted RNAs were analyses by quantitative real time PCR as described previously. The primers used in this study are listed in Table 1.

#### Detection of Virus Replication

RTgill-W1 cells were grown onto 12-well transwells and cells were allowed to grow until confluency. Cells were then infected with SAV2 at MOI 10, 1, 0.1, and 0 for 6, 12, 18, 24, and 96 h. Each treatment was conducted in triplicate and cells from each treatment group were pooled for cytoplasmic RNA extraction. As SAV-2 replication occurs in the cytoplasm, cytoplasmic RNA was used for the quantification of viral load.

To detect the replicative strand in RTgill-W1 cells, cytosolic RNA was used for strand specific RT-qPCR (32). A tailed SAV-2 primer with a specific tag was used for cDNA synthesis using superscript III kit (Invitrogen) using primer SAVFPtag (5′-ggccgtcatggtggcgaattccac caccccgaagaagtc-3′). For the qPCR detection of SAV-2 replicative strand primer SSTag (5′-ggccgtcatggtggcgaat-3′) complementary to the sequence introduced by the tailed primer was used (PCR temperature profile: reverse transcription at 95°C/10 min, initial denaturation at 95°C/3 min and 55 cycles of PCR at 95°C/10s, 58°C/20s, 72°C/15s, and cooling 40°C/30s). Ramping rate was the same as mentioned earlier.

### Poly(I:C) Induced Phosphorylation in RTgill-W1 Cells

#### Cell Culture, Stimulation, and Cell Lysis

RTgill-W1 cells were grown onto 6-well transwells with a membrane pore size of 0.4 μm and a growth area of 4.2 cm^2^. Cells were seeded at a density of 0.8 × 10^6^ cells/transwell and were maintained until they reached confluency. Cells were stimulated with poly(I:C) at a concentration of 10 μg/ml for 30 min. After 30 min of stimulation, cells were washed three times with PBS followed by lysis with 400 μl lysis-buffer [1% (w/v) Sodium deoxycholate in 50 mM ammonium bicarbonate and 1% phosphatase inhibitor cocktail (v/v) (Thermo Fisher Scientific, Hemel Hempstead, UK)] per insert. Lysates from 4 inserts from each treatment (control and polyI:C treated cells) were pooled. A total of *n* = 4 control samples and *n* = 5 poly(I:C) treated samples were generated and analyzed as replicates.

#### Protein Quantification and Visualization by 1D SDS-PAGE

Protein quantification was performed using BCA assay (Interchim Uptima, France) using Bovine Serum Albumin (BSA) (stock 2 mg/ml) as standard. To check the quality and integrity of the cell protein lysates 10 μg of the extracted proteins from each sample were visualized by 1-Dimensional Sodium Dodecyl Sulfate Polyacrylamide Gel Electrophoresis (1D SDS-PAGE gel).

#### Trypsin Digestion, Clean-Up, and Peptide Enrichment

Protein samples (1 mg/sample) were dried in Savant DNA 110 SpeedVac® Concentrator and re-suspended in 400–500 μl of 2 mM DTT (Dithiothreitol) (Bio-Rad, UK) in 50 mM ammonium bicarbonate (Sigma-Aldrich, UK) by vortexing until protein dissolved completely. Samples were incubated for 60 min at 37°C followed by another incubation for 60 min at 37°C in dark after adding 5 μl/100 μl of freshly prepared iodoacetamide, IAA (200 mM) (Fisher Scientific, Leicestershire, UK). Then 10 μl of DTT (200 mM) was added followed by further incubation for 30 min at room temperature in dark. Finally, sequencing grade modified trypsin (Promega, UK) was added in a trypsin to protein ratio of 1:100 and incubated at 37°C overnight in the dark. On the following day, protein digestion was stopped by adding 96% formic acid (Fisher Scientific, Leicestershire, UK) to a final concentration of 1%. Clean-up of digested samples was performed using HyperSep™ SpinTips (Thermo Fisher Scientific, Hemel Hempstead, UK) according to the manufacturer's instruction. Peptide samples were eluted in 50 μl of releasing solution [40% of 0.1% formic acid in miliQ water plus 60% of acetonitrile (Fisher Scientific, Leicestershire, UK)]. Eluted samples were directly dried in SpeedVac concentrator for enrichment or stored at 4°C until further usage. Phosphopeptide enrichment was done using High-Select TiO_2_ Phosphopeptide enrichment kit (Thermo Scientific, Hemel Hempstead, UK) following the manufacturer's instructions. Eluted phosphopeptides were dried and stored at −80°C until analysis by LC-MS/MS.

#### LC MS/MS

Enriched phosphopeptide samples were analyzed using a Dionex Ultimate 3000 RSLS nano-flow system (Dionex, Camberley UK). Samples were reconstituted in 10 μL of water and a volume of 5 μL were loaded onto a Dionex 100 μm × 2 cm 5 μm C_18_ nano-trap column at a flow rate of 5 μL min^−1^. The composition of the loading solution was 0.1% formic acid and ACN (98:2). Once loaded onto the trap column samples were washed off into an Acclaim PepMap C_18_ nano-column 75 μm × 15 cm, 2 μm 100 Å at a flowrate of 0.3 μL min^−1^. The trap and nano-flow column were kept at 35°C in a column oven in the Ultimate 3000 RSLC. Samples were eluted with a gradient of solvent A: 0.1% formic acid and ACN (98:2) vs. solvent B: 0.1% formic acid and ACN (20:80) starting at 5% B and increasing to 50% B over 100 min. The column was washed using 90% B before being equilibrated prior to the next sample being loaded. The eluant from the column was directed to a Proxeon nano-spray ESI source (Thermo Fisher, Hemel, UK) operating in positive ion mode then into an Orbitrap Velos Fourier Transform (FTMS). The ionization voltage was 2.5 kV and the capillary temperature was 200°C. The mass spectrometer was operated in MS–MS mode scanning from 380 to 2000 amu. The top 20 multiply charged ions were selected from each full scan for MS/MS analysis, the fragmentation method was HCD at 30% collision energy. The ions were selected for MS2 using a data dependant method with a repeat count of 1 and repeat and exclusion time of 15s. Precursor ions with a charge state of 1 were rejected. The resolution of ions in MS 1 was 60,000 and 7500 for MS2.

#### MS Data Processing

LC-MS/MS data were processed initially uploading the raw spectra data into Thermo Proteome Discoverer 1.4 (Thermo Scientific, Hemel Hempstead, UK). Peak picking was performed under default settings for FTMS analysis such that only peptides with signal to noise ratio higher than 1.5 and belonging to precursor peptides between 700 and 8,000 Da were considered. Peptide and protein identification were performed with SEQUEST algorithm. An in house compiled database containing proteins from the latest version of the UniProt SwissProt database (2017) was compiled to include only *Oncorhynchus mykiss*. The search parameters were: Tryptic cleavage with 2 missed cleavages; static modification was carbamidomethyl of cysteines; allowed dynamic modifications were oxidation of methionine and phosphorlyation of serine, threonine, and tyrosine. Precursor tolerance was set at 10 ppm and MS2 tolerance was set at 0.05 Da. Resulting peptides and protein hits were further screened by excluding peptides with an error tolerance higher than 10 ppm and by accepting only those with an FDR<0.05. Protein identification was based on the presence of at least one unique peptide and quantification was based exclusively on unique peptide(s).

#### Gene Ontology (GO) Enrichment Analysis and Sub-cellular Localization of Phosphoproteins

*Enrichr* (http://amp.pharm.mssm.edu/Enrichr (33, 34) tool was used to perform GO. Since the tool supports only human, mouse and rat genes, best matched human homologs of the trout genes identified in rainbow trout were used. To this end, trout protein accession numbers were blasted in NCBI against zebrafish (*Danio rerio*) and zebrafish uniprot protein IDs were exported. The gene symbols of respective proteins of zebrafish were then extracted using the biological Database network (https://biodbnet-abcc.ncifcrf.gov/db/db2db.php). Finally, zebrafish gene symbols were then converted to human gene symbols using OrthoRetriever tool (http://lighthouse.ucsf.edu/orthoretriever/). Adjusted p-value and combined score were considered for GO annotation and kinase prediction where the combined score was described by Chen et al. (33) as c = log(p) × z, where c = the combined score, p = Fisher exact test p-value, and z = z-score for deviation from expected rank. For general cellular and molecular characterization of phosphoproteins identified in rainbow trout gill epithelia in control and treatment conditions, all the phosphoproteins identified in each group were used for GO analysis. Kinase enrichment analysis was also performed using all the phosphoproteins in each group. Predicted sub-cellular localization of phosphoproteins was obtained using WoLF PSORT algorithm (https://wolfpsort.hgc.jp) based on both known signal motifs and their amino acid sequence features having over 80% prediction accuracy (35).

#### Phosphorylation Motif Analysis and Kinase Identification

Phosphopeptide sequences were uploaded to pLogo algorithm (https://plogo.uconn.edu) for the identification motifs present in each data set. Sequences were centered on each phosphorylation site and extended to a total length of 15 amino acids (±7 residues) using an *ad hoc* Microsoft Excel routine. When the site was located in the N/C-terminal of the protein, the sequence was filled up to 15 amino acids with the required number of “X” (missing amino acid positions). The sequences of proteins from rainbow trout identified in this study were used as a background dataset. For the graphical presentation of the identified motifs, logo-like representations were generated for each motif using pLogo based on their statistical significance (*p* < 0.05).

#### Pathway Analysis

The gene symbols of human counterparts of the identified rainbow trout proteins were used in *Enrichr* and significantly enriched (*p* < 0.05) KEGG pathways were predicted. Relevant KEGG pathways for human were exported and the pathways were adopted for the genes identified in rainbow trout using the tool *Pathvisio* (https://www.pathvisio.org/); (36). Similar to GO annotation and KEA, all the phosphoproteins identified in each group were used for pathway analysis to have overall picture regarding the signaling pathways activated in steady state and stimulated RTgill-W1 cells.

### Statistical Analysis

TEER data were analyzed using 2-way (treatments and durations as factors) repeated measure ANOVA followed by Bonferoni's multiple comparison using GraphPad prism version 6.0 (GraphPad Software) (San Diego, CA, USA). RT-qPCR data were analyzed using one-way ANOVA followed by Bonferroni's multiple comparison using GraphPad prism version 6.0 (San Diego, CA, USA). In all analyses, differences between groups were considered statistically significant at *p* < 0.01 unless otherwise stated. For phosphoproteomics, sequences of uncharacterised proteins were blasted in Protein BLASTp of NCBI (https://blast.ncbi.nlm.nih.gov/Blast.cgi); (37). The protein homologies were selected according to the criteria (identity >80%, E value <0.001) demonstrated by Pearson (38). Phosphoproteins were visualized by Venn diagram using online tool *BioVenn* (http://www.biovenn.nl); (39). Shared phosphoproteins were further analyzed by t-test (*p* < 0.05) and represented by volcano plot using RStudio version 1.0.153.

## Results

### Changes of Epithelial Integrity in Response to Poly(I:C)

**Figure 1:**
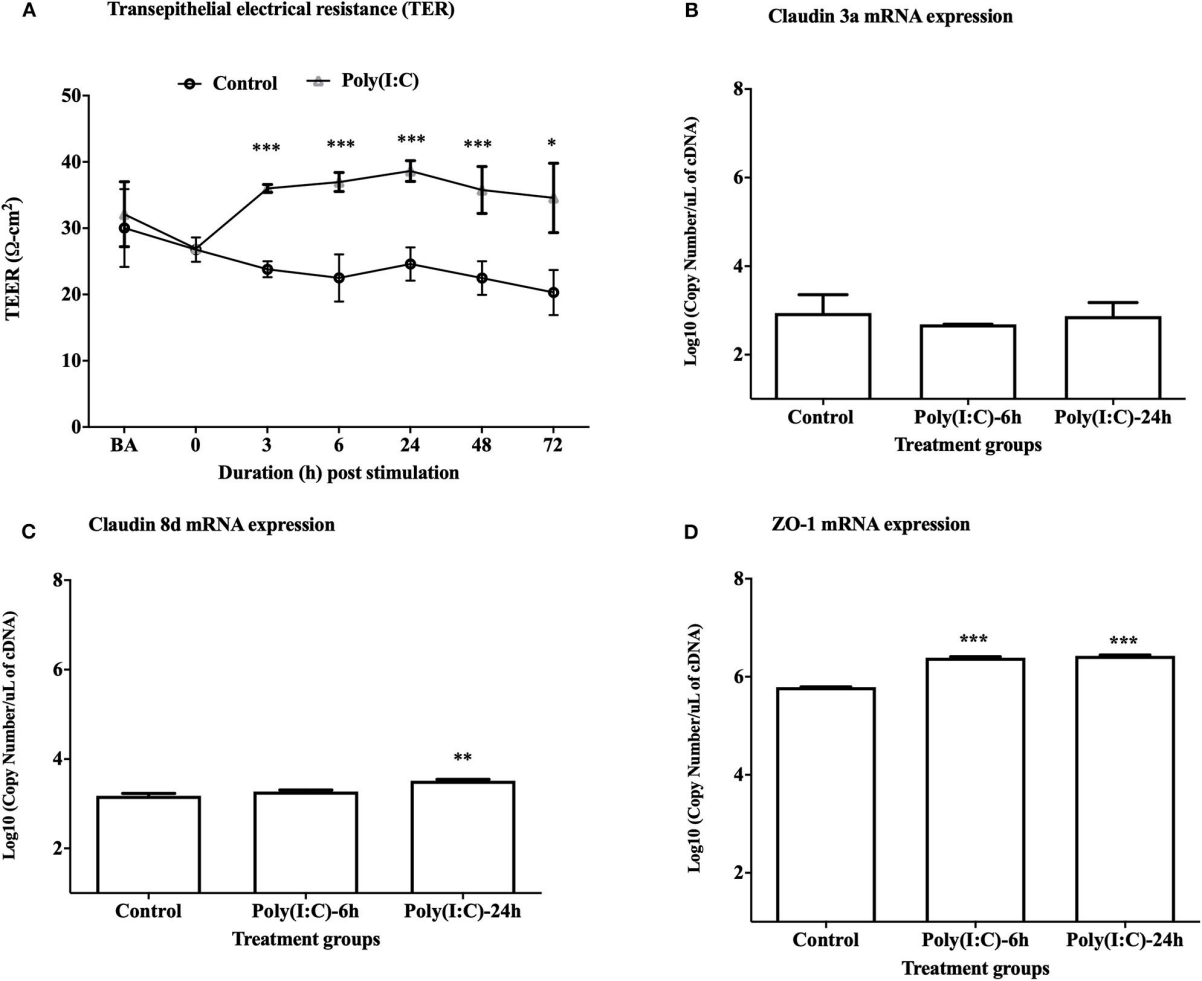
Transepithelial electrical resistance (TEER) and tight junction gene expression. **(A)** Effect of dsRNA on transepithelial electrical resistance (TEER) across RTgill-W1 cell layers where 10 μg/ml poly(I:C) was used to stimulate the cells. Cells were stimulated at nearly 72 h after seeding in transwells when TEER was stable. Any course 3 independent experiments in triplicate were conducted having 3 measurements at a single time point in each insert. Values are mean ± SEM (*n* = 3 × 3 = 9). Repeated measure one-way ANOVA was used to analyse the data at *p* < 0.01. BA stands for “before activation”. **(B–D)** Tight junction gene claudin 3a, 8d and ZO-1 expression upon stimulation with poly(I:C) at a concentration of 10 μg/ml for 6 and 24 h in the transwells. Data are mean ± SEM of three course independent experiment. One-way ANOVA followed by Bonferoni's multiple comparison was used to analyse the data with the level of significance between control and treatment at *p* < 0.01. *p < 0.01, **p < 0.001, ***p < 0.0001. No asterisk indicates lack of significant differences at a specific time point.

To study the cellular integrity of RTgill-W1 cells we monitored transepithelial resistance (TEER). Cells were grown in transwells and post-seeding TEER was measured at different time points where maximum average TEER (around 30 and 32 Ω-cm^2^, respectively, in two groups for control and stimulated cells as indicated by BA in Figure 1A) was recorded at 48 h post seeding. TEER was then measured every 6 h until a stable TEER observed. At 72 h post seeding a stable TEER was observed which was around 26 Ω-cm^2^ (indicated at time 0 in Figure 1A). Immediately after stimulation with poly(I:C) at 10 μg/ml, TEER increased until 24 h in stimulated cells and then decreased slowly at 48 and 72 h post stimulation (Figure 1A). TEER was found to return to baseline values after 6–7 days of stimulation in preliminary trial experiments (data not shown). Significantly higher TEER value was detected in poly(I:C) stimulated cells than in control cells at 3 h post stimulation and onwards (*p* < 0.001). To investigate the effects of poly(I:C) on the epithelial integrity of RTgill-W1 cells at a molecular level, the expression of a set of tight junction related genes was analyzed. Poly(I:C) at 10 μg/ml was not found to stimulate the mRNA expression of claudin 3a (Figure 1B). However, Claudin 8d mRNA at 24 h and ZO-1 mRNA at 6 and 24 h were slightly but significantly upregulated in RTgill-W1 cells stimulated with poly(I:C) at 10 μg/ml (*p* < 0.001 and *p* < 0.0001, respectively) (Figures 1C,D).

### TLR3 Mediated Innate Immune Response Upon Poly(I:C) Stimulation

**Figure 2:**
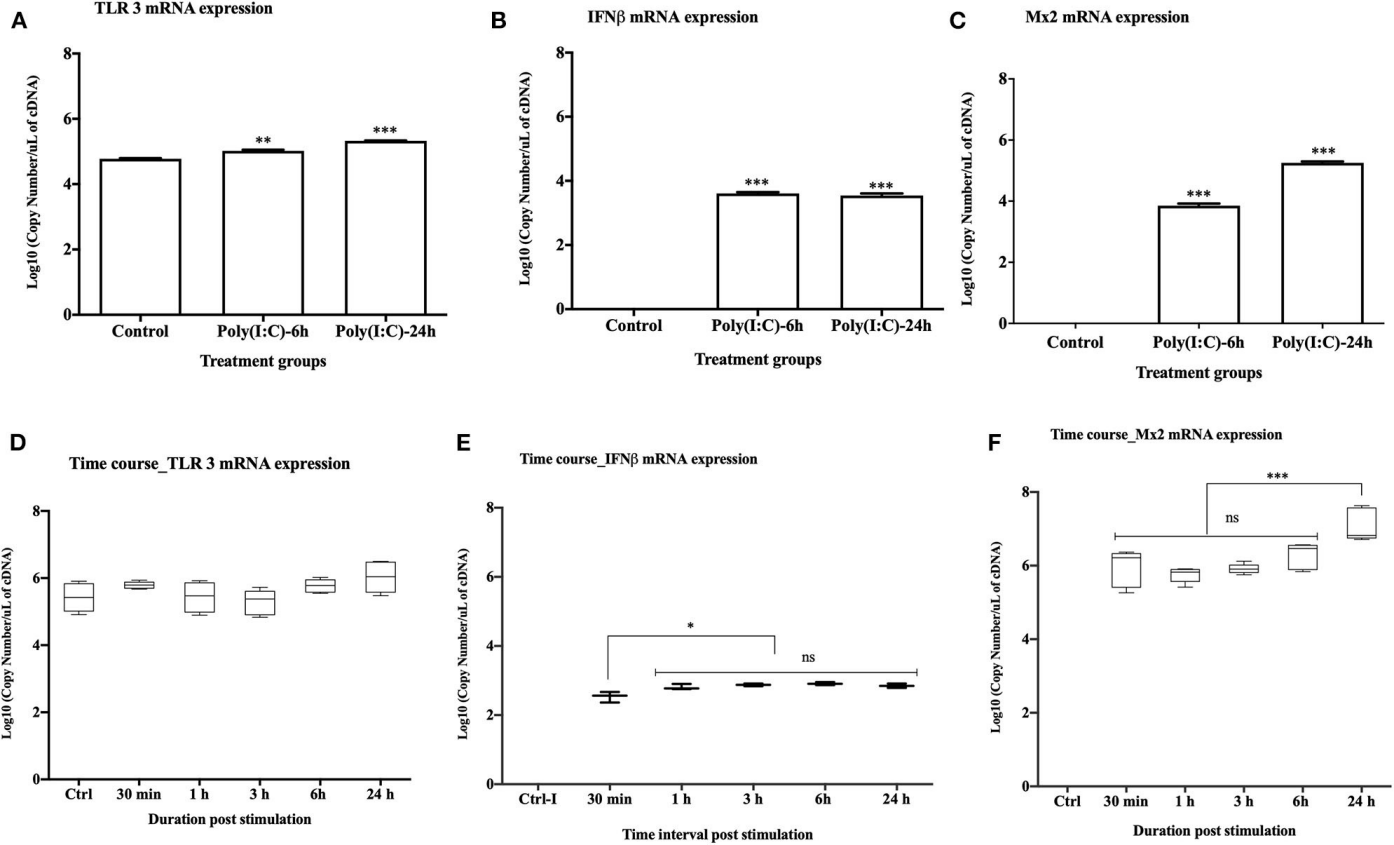
Antiviral response of RTgill-W1 cells. **(A–C)** TLR3, rtIFN2, and Mx2 expression upon stimulation with poly(I:C) at a concentration of 10 μg/mL for 6 and 24 h in the transwells where data were compared between control and treatment. **(D–F)** Time course response of RTgill-W1 cells upon stimulation with poly(I:C) at a concentration of 1 μg/ml. Data show mean ± SEM of three course independent experiment. One-way ANOVA followed by Bonferoni's multiple comparison was used to analyse the data with the level of significance at *p* < 0.01. *p < 0.01, **p < 0.001, ***p < 0.0001 and ns, not significant.

Upon stimulation with poly(I:C) at 10 μg/ml, TLR3 mRNA expression was significantly upregulated at both 6 and 24 h post stimulation (*p* < 0.001 and *p* < 0.0001, respectively) (Figure 2A). Expression of rtIFN2 and Mx2 mRNAs was detected at 6 and 24 h post stimulation but not in control group (Figures 2B,C). To determine the time-point of initiation of the antiviral response, a more detailed time course response experiment was conducted where cells were stimulated with poly(I:C) for 0.5, 1, 3, 6, and 24 h using a lower poly(I:C) dose (1 μg/ml). In this experiment, a steady expression of mRNA transcripts of TLR3 was found throughout the experiment (Figure 2D), while a very low level of expression of rtIFN2 mRNA was detected. However, mRNA transcript levels of rtIFN2 were detected as early as 30 min post- stimulation and levels further increased after 3, 6, and 24 h post stimulation (*p* < 0.01) (Figure 2E). Similarly, *de novo* mRNA expression of the antiviral response gene, Mx2, initiated at 30 min of poly(I:C) stimulation with a progressive increase up to 24 h of stimulation (*p* < 0.0001) (Figure 2F).

### SAV-2 Replication in RTgill-W1 Cells

SAV-2 was used to investigate the cellular and molecular response of RTgill-W1 cells upon viral infection. To optimize SAV culture yield three cell lines were tested and cytopathic effect (CPE) was found at days 5, 3, and 6 in CHSE-214, CHH-1 and TO cells, respectively, following inoculation by the adsorption method (data not shown). In cells inoculated using the simultaneous method, CPE started to form at day 4, 4, and 6 for all three cell lines, respectively. Although, CPE developed earlier in the CHH-1 cell line, CPE was more progressive in CHSE-214 cells. Virus was harvested at day 18 from all the cells and quantified by real time PCR. The highest viral RNA copy number was found in CHSE-214 cell inoculated by adsorption with a viral stock of 10^−3^ dilution.

**Figure 3:**
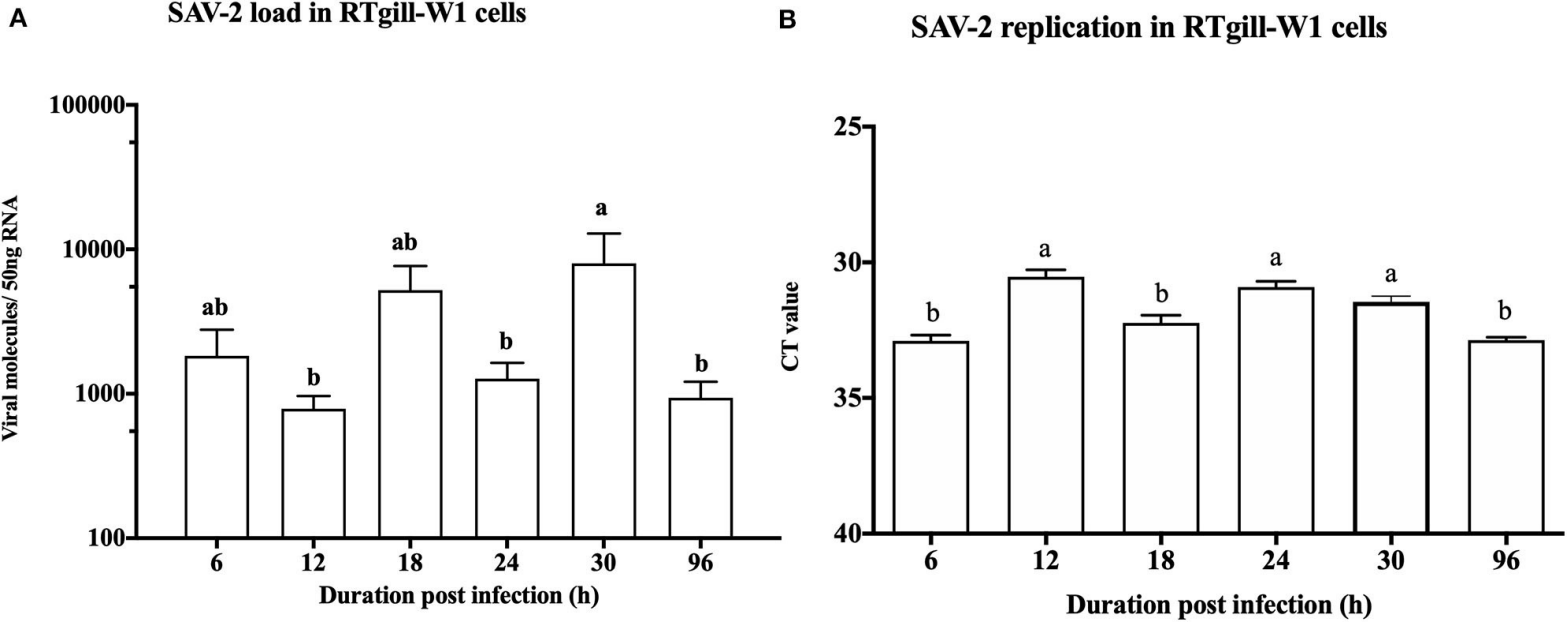
Viral load **(A)** and replication **(B)** in RTgill-W1 cells at different time points of post infection with SAV-2 at MOI-10. Viral copy number was determined using Taqman probe-based RT-qPCR and SAV-2 replicative strand was detected using strand specific RT-qPCR. Values were mean ± SEM. Bars with different letters are significantly different with a level of significance at *p* < 0.01. One-way ANOVA followed by Bonferoni's multiple comparison was used to analyse the data.

To monitor the viral genome load of SAV-2 in RTgill-W1 cells in a time dependent manner cytosolic RNA was extracted and tested by TaqMan probe-based RT-qPCR for SAV-2. A low viral copy number was detected in the cytosolic RNA throughout the experimental period (Figure 3A). A significantly higher viral copy number was detected at 30 h post infection (p<0.01). To confirm replication of SAV-2 in RTgill-W1 cells, cells were infected with SAV-2 at MOI-10, 1 and 0.1 and cytosolic RNA extracts tested using strand specific RT-qPCR. SAV-2 was found to replicate in all cells tested. Virus replication inside the RTgill-W1 cells was detected as early as 6 h post infection (Figure 3B). However, viral replication was significantly higher at 12 and 24 h post infection (*p* < 0.01) in cells infected at MOI-10. However, viral load and replication in cells infected at MOI-1 and MOI-0.1 were very low (data not shown).

### Cellular Response of RTgill-W1 Cells Upon SAV-2 Infection

**Figure 4:**
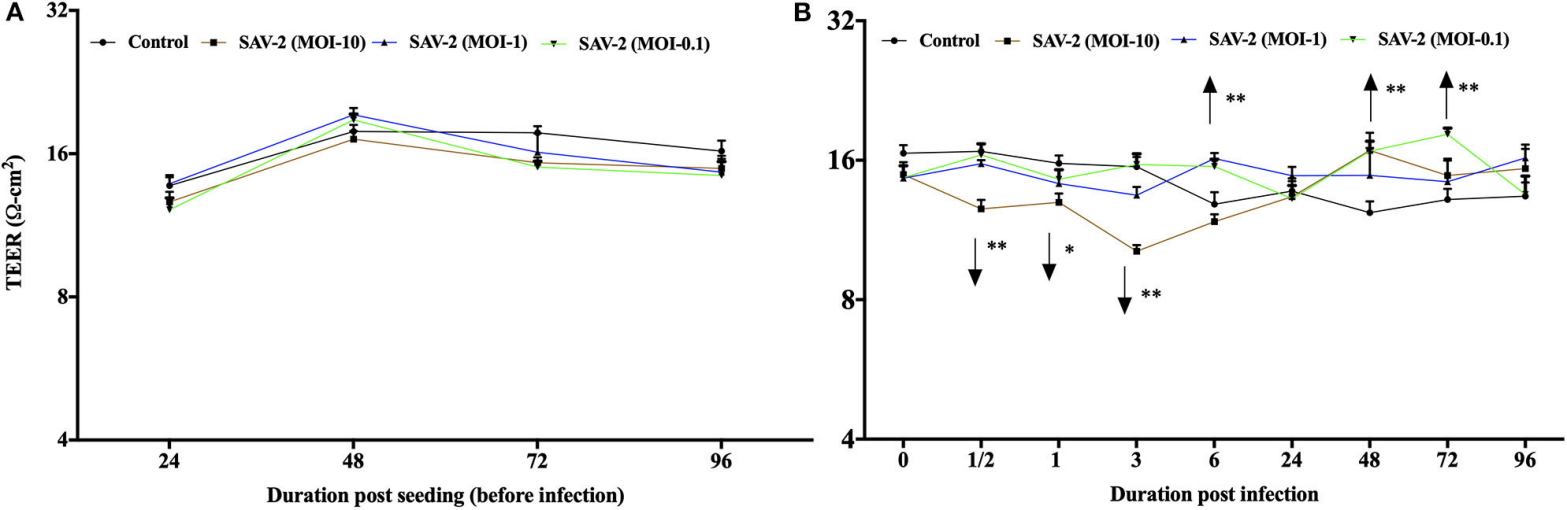
Modulation of transepithelial electrical resistance (TEER) of RTgill-W1 cells. **(A)** Cells were seeded onto the transwells and left uninfected until TEER remained stable. **(B)** TEER in response to SAV-2 infection at MOI-10, 1, and 0.1 at different time points. In all cases 3 independent experiments in triplicate were conducted with 3 measurements at a single time point in each replicate. Values show mean ± SEM (*n* = 3 × 3 = 9). TEER of infected groups was compared to control group in each time point using repeated measure one-way ANOVA with the level of significance at *p < 0.01 and **p < 0.001.

To investigate the effects of SAV-2 RTgill-W1 cells were infected with SAV-2 at MOI-10, 1 and 0.1. Before SAV-2 infection, TEER in each group was monitored until it remained stable (16–20 Ω.cm^2^) at 72 to 96 h (Repeated measure one-way ANOVA; Figure 4A). Post infection TEER in the MOI-10 infected group started to decrease from as early as 0.5 h until 3 h post infection (hpi) and then increased significant at 48 hpi (*p* < 0.001). On the other hand, in the MOI-1 and 0.1 infected groups, TEER was similar to the control group until 3 hpi (Figure 4B). At 6 hpi, significantly higher TER was measured in MOI-1 infected group than the control group (*p* < 0.001). Moreover, TEER measured in RTgill-W1 cells of MIO-0.1 group was significantly higher than the control group at 48 and 72 hpi (*p* < 0.001).

### SAV-2 Mediated Antiviral Response Through TLR3 Signaling Pathway

**Figure 5:**
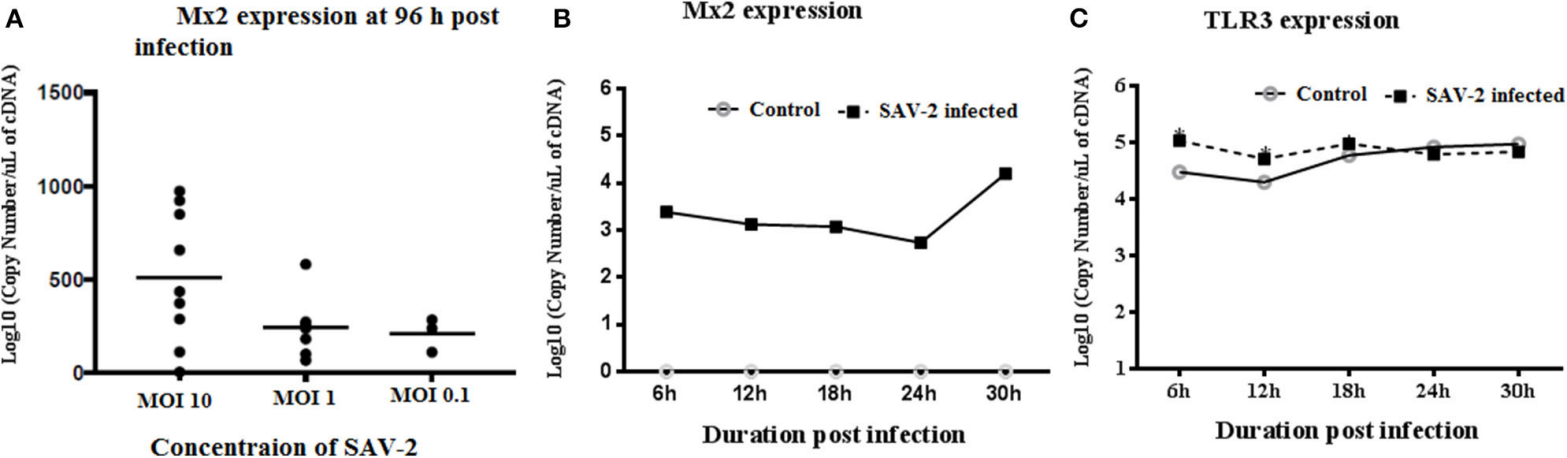
Expression profile of TLR3 and Mx2 in the cytoplasmic RNA of RTgill-W1 cells. **(A)** Expression of Mx2 at 96 h of post infection with SAV-2 at MOI−10, 1 and 0.1, **(B)** expression of Mx2 at different time points infected with SAV-2 at MOI−10 and **(C)** TLR3 expression at different time points infected with SAV-2 at MOI-10 were measured. One-way ANOVA followed by Bonferoni's multiple comparison was conducted to analyse the data where statistical significance was determined at *p* < 0.01.

To investigate the transcriptomic response of RTgill-W1 cells to viral infection we monitored the expression of a set of mRNAs relevant to; (1) tight junction regulation including ZO-1, claudin 3a and 8d, and 2) antiviral signaling including TLR3, Mx2 and rtIFN2. SAV-2 did not induce the expression of the tight junction regulatory gene ZO-1, nor claudin 3a and 8d (data not shown). Expression of the interferon stimulated gene, Mx2 indicated an antiviral response in infected cells which correlated to the viral input concentration at 96 h post infection. Expression of Mx2 mRNAs in MOI-10 infected cells was around 2.5-fold higher than that in MOI-0.1 infected cells (Figure 5A). The time course of Mx2 mRNA abundance was monitored at 6 h intervals in cytoplasmic and total RNA fractions in control and SAV-2 infected cells (MOI-10) where only infected cells showed the expression. The highest abundance of Mx2 mRNA transcripts was observed at 30 h post infection with a significantly higher Mx2 mRNA expression than all other time points measured (*p* < 0.0001; Figure 5B). In comparison, the expression of the endosomal dsRNA sensor, TLR3 upon SAV-2 infection with MOI-10 only showed significantly higher copy numbers at early stages of infection (6–12 h) (*p* < 0.001; Figure 5C). rtIFN2 mRNA copy number remained stable but low throughout the experiments (data not shown).

### Antiviral Response Through RLR Signaling Pathway

**Figure 6:**
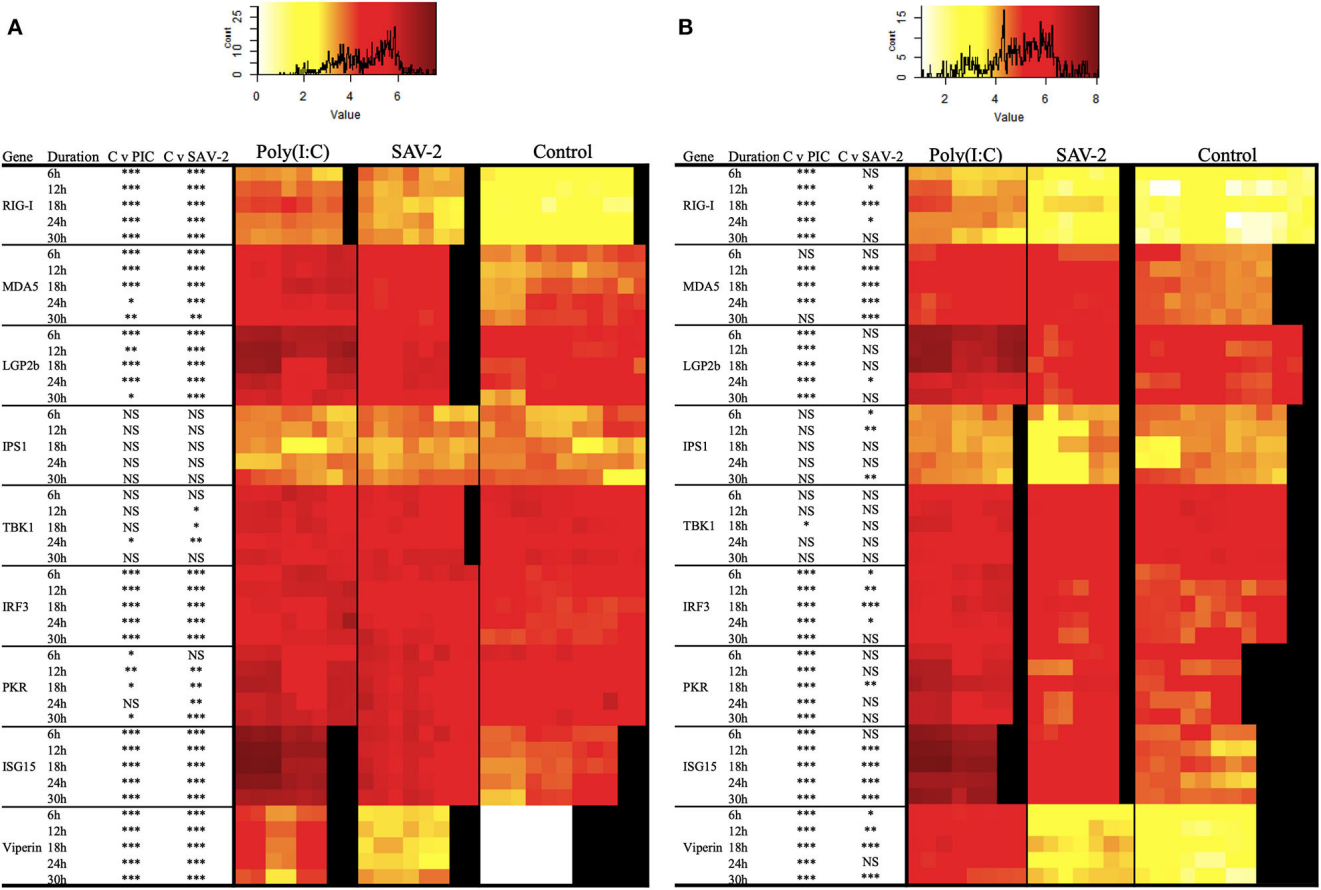
Expression profiles of mRNA transcripts for RLRs and integration and effector molecules in RT-gill-W1 cells. Heatmaps showing the absolute quantification values of mRNA transcripts in cytoplasmic **(A)** and total **(B)** RNA fractions in RT-gill-W1 cells. Color scales indicate the relationship between colors and absolute mRNA values. Significant upregulation at *p* < 0.0001, *p* < 0.001, and *p* < 0.01 are expressed by the asterisk ***, ** and *, respectively; NS indicates no significance. Each square in the heatmaps represents each sample tested. Black color in the heatmaps represents no sample. PIC stands for poly(I:C).

To further investigate the regulation of dsRNA sensing and the activation of RLR signaling the expression of several genes associated with the pathway were screened. Double stranded RNA was found to stimulate an increased mRNA copy number of all of the tested receptor molecules, RIG-I, MDA5, and LGP2b, at all the time points tested for both cytoplasmic and total RNA (*p* < 0.0001). The exception was for MDA5 mRNA copy numbers at 6 and 30 h post stimulation in cytoplasmic RNA and 30 h of post stimulation in total RNA (Figures 6A,B). Signaling molecules, IPS1 and TBK1, remained stable upon stimulation with poly(I:C) while expression of IRF3 in both mRNA fractions was significantly and constitutively upregulated upon stimulation (Figure 6). All interferon stimulated genes (ISGs) including PKR, ISG15 and viperin were significantly upregulated in both cytoplasmic and total RNA upon stimulation with poly(I:C) (*p* < 0.0001).

To assess whether SAV-2 also triggers the RLR pathway, the above transcripts were also measured upon SAV-2 infection. In the cytoplasmic RNA, RIG-I mRNA transcript were significantly and constantly upregulated in comparison to control cells at all time points (*p* < 0.0001). In total RNA, RIG-I was upregulated at 12 and 24 h post infection in response to SAV-2. Expression of MDA5 in the cytoplasmic RNA was upregulated at the early stage of infection until18 h post infection and remained unchanged at the later stage of SAV-2 infection in both cytoplasmic and total RNA. LGP2b, a splice variant of LGP2, mRNA copy number was also upregulated in the cytoplasmic fraction in all the time points (*p* <0.0001) while in the total RNA fraction LGP2b was upregulated only at 24 h post infection (*p* < 0.01). In cytoplasmic RNA, IPS1 copy numbers remained stable in both control and SAV-2 infected cells throughout the experiment. However, in the total RNA fraction, IPS1 expression was downregulated at the early stage of infection at 6 h (*p* <0.01) and 12 h (*p* < 0.001) which returned to basal levels at 18 h and was further downregulated at 30 h (*p* < 0.001) post infection. TBK1 in the cytoplasmic fraction was slightly but significantly upregulated at 12 (*p* < 0.01), 18 (*p* < 0.01), and 24 (*p* < 0.001) h post infection. Interestingly, when using a total RNA fraction, TBK1 expression was unaffected by SAV-2 infection. For IRF3, in the cytoplasmic RNA fraction, there was a constitutive and significant upregulation following SAV-2 infection whilst in total RNA, expression was upregulated at 18 h post infection (*p* < 0.0001). Both ISG15 and viperin mRNA abundances were significantly upregulated at all the time points in SAV-2 infected cells in the cytoplasmic RNA fraction (*p* < 0.0001). In the total RNA fraction, ISG15 was upregulated at 12 h of infection and onwards while viperin expression was upregulated at 6 h of SAV-2 infection and onwards.

### Phosphoproteome Analysis of RTgill-W1 Cells

**Figure 7:**
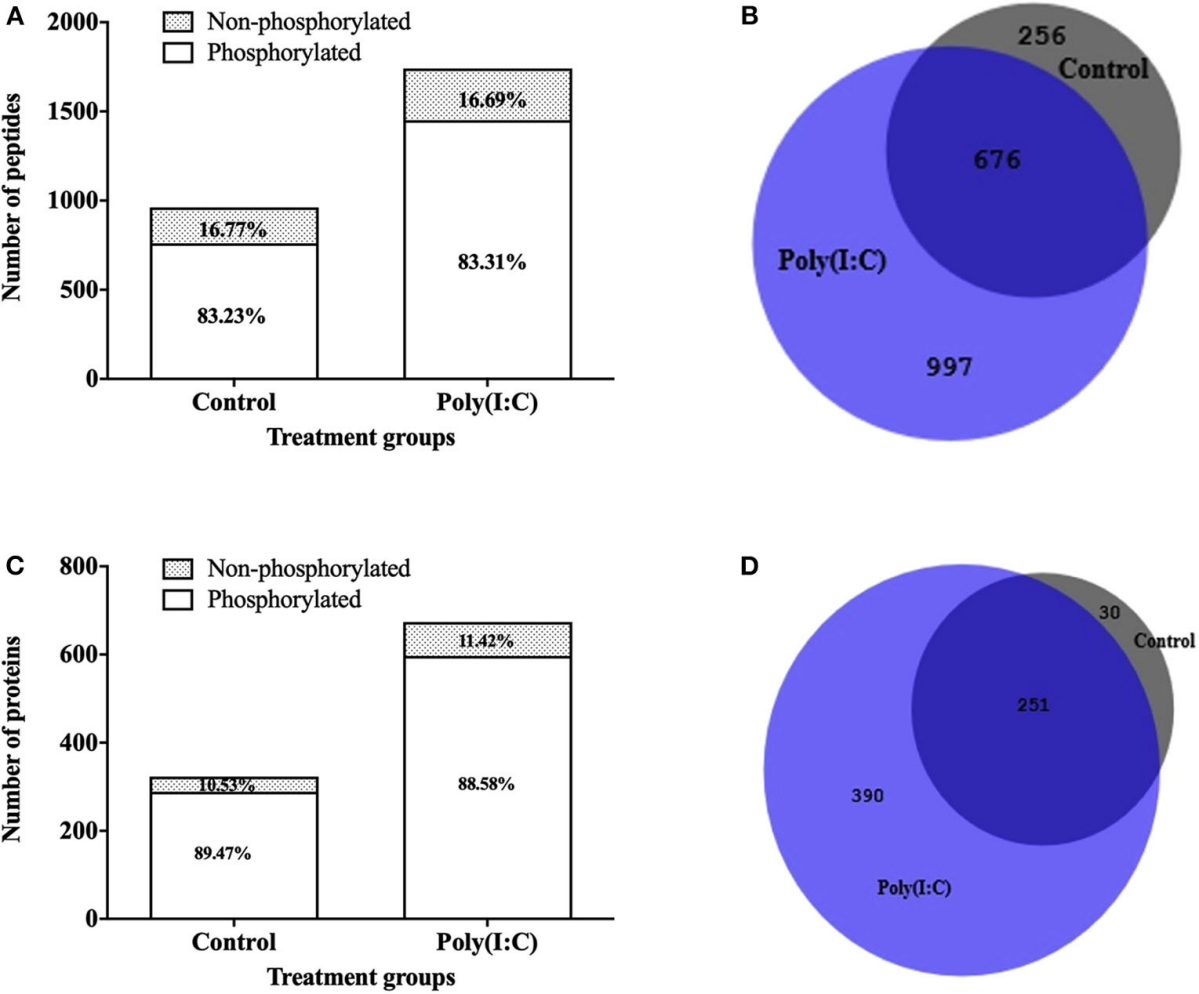
Phosphopeptides identified in RTgill-W1 cells in different treatment groups. **(A)** Stacked histogram showing the number and percentage of phosphopeptides and non-phosphopeptides. **(B)** Venn diagram showing the number of phosphopeptides shared and unique between the different treatment groups. Number of phosphoproteins identified in RTgill-W1 cells in different groups. **(C)** Actual number of proteins in control and poly(I:C) stimulated cells. **(D)** Venn diagram showing the distribution of phosphoproteins in different groups.

To study the signaling mechanisms involved in antiviral immune response, the phosphoproteome of RTgill-W1 cells was analyzed by LC-MSMS following phosphopeptide enrichment in control and in cells stimulated with poly(I:C). Phosphopeptide enrichment, yielded a similar percentage of phosphorylated peptides in all samples (83.23 ± 7.58 and 83.30 ± 5.33% in control and poly(I:C) stimulated cells, respectively) (Figure 7A). However, the actual number of phosphopeptides was higher in poly(I:C) stimulated cells (1,671 phosphopeptides, compared to 932 for the control group) although as already mentioned the percentage of phosphopeptide enrichment was similar. In total, 1,929 phosphopeptides were identified, 799 peptides of which were unique to poly(I:C)-stimulated cells (Figure 7B). A total of 2,612 phosphorylation sites were detected in 1,929 phosphopeptides. More than half of the phosphopeptides were monophosphopeptides (65%) whereas 30% were diphosphopeptides. The distribution of phosphorylated amino acids was similar in all samples, ranging from 70.72 to 73.80% pSer, 20.05–21.55% pThre and 5.91–7.23% pTyr. Phosphopeptide identifications were compiled at protein level and resulted in the identification of a total of 671 phosphoproteins. Poly(I:C) stimulated cells had 641 phosphoproteins, 390 of which were unique (Figures 7C,D). Information on the phosphoproteins identified is available in Supplementary Table 1.

### Gene Ontology (GO) Enrichment Analysis and Sub-cellular Localization of Phosphoproteins

**Figure 8:**
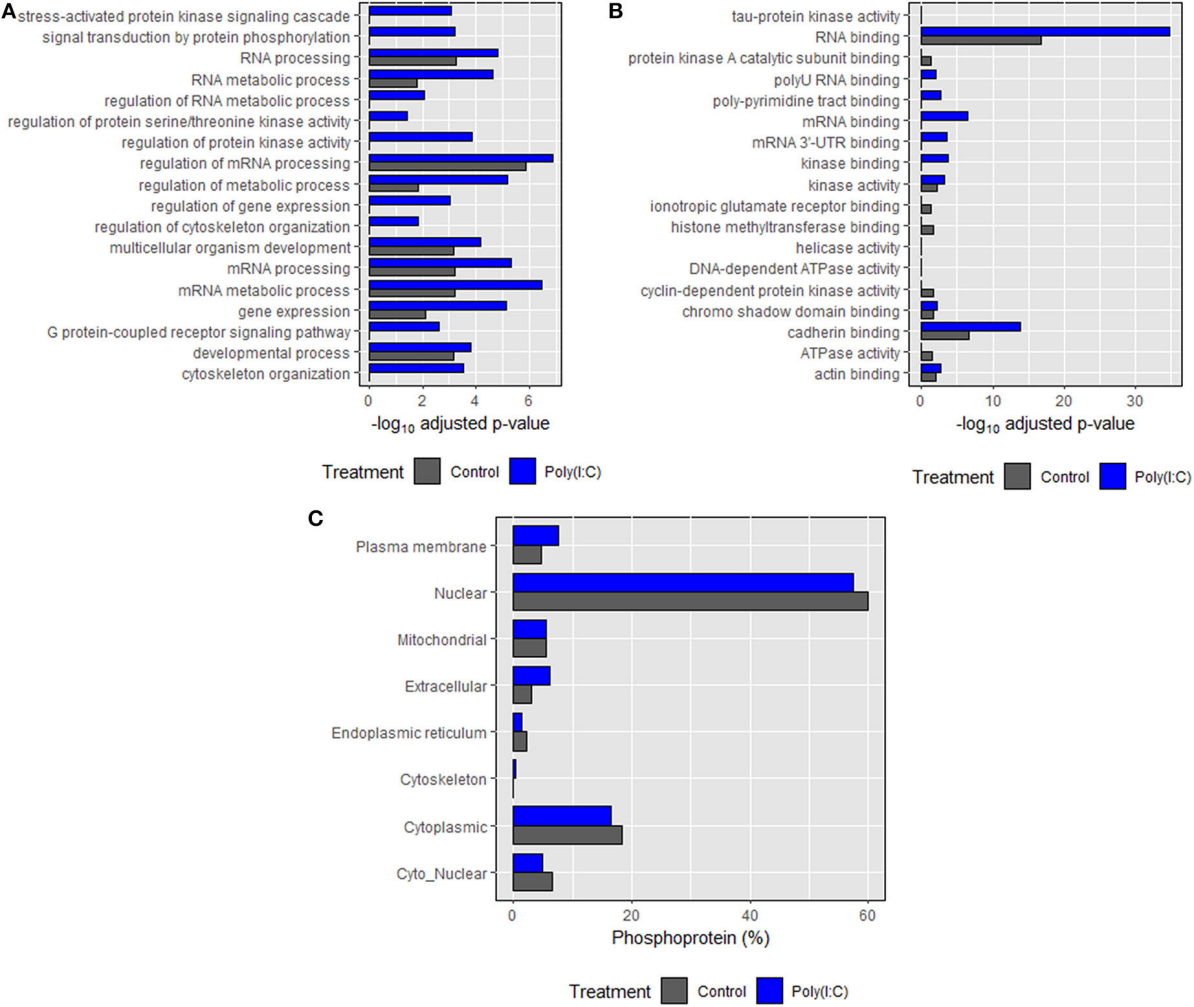
Gene ontology (GO) enrichment analysis of the identified proteins. **(A)** Biological processes, **(B)** molecular function, and **(C)** subcellular localization of phosphoproteins identified in control and poly(I:C) stimulated cells sharing each component of GO. Biological processes and molecular function were predicted using *Enrichr* tool while subcellular localization was predicted using WoLF PSORT algorithm. Top 10 entries of each category (on the basis of combined score, which has been given by the tool) were then plotted against the negative logarithm of adjusted *p* values (*p* < 0.05).

Based on annotations obtained according to biological processes, an enrichment in phosphoproteins associated with RNA and mRNA processing, regulation, and metabolism was induced. Many of the biological processes such as cytoskeleton organization, signal transduction, regulation of cytoskeleton organization and protein kinase activity were found only in the poly(I:C) stimulated cells (Figure 8A). In the case of molecular functions, an enrichment in binding and receptor activity including RNA binding was found. Transmembrane signaling receptor and G-protein coupled receptor activity related proteins were found only in poly(I:C)-stimulated cells (Figure 8B). In most cases, significantly higher enrichment was found in poly(I:C) stimulated cells compared to control cells. For both groups, most of the phosphoproteins were predicted to be localized in the nucleus and cytoplasm, which comprised around 80% of total phosphoproteins identified (Figure 8C). Phosphoproteins localized in the cytoskeleton were found only in poly(I:C)-stimulated cells. Similarly, the percentage of phosphoproteins residing in the extracellular and plasma membrane was higher in poly(I:C) stimulated cells compared to control cells. Full data are available in Supplementary Data Sheet 1.

### Motif Analysis and Kinase Prediction

To find significant motifs in the phosphopeptide data, firstly, overall motifs were identified for each group for serine and threonine residues (Supplementary Figure 1). Individual motifs were then extracted from the overall motifs for serine and threonine residues for each group (Individual plogo figure not shown). For serine residues, a total of 81 different motifs were detected of which 43 were unique to poly(I:C) treated cells and 7 were unique to control cells (Supplementary Table 1). For threonine residue, only 4 motifs were identified with 2 being unique to poly(I:C) treated cells. No unique motif for phosphopeptides in control cells for threonine residue was identified. No significant motifs were found for phosphotyrosine residue in any of the groups. Associated kinases for the motif substrates were identified by literature search (Supplementary Table 1) with the caveat that predicted kinases were based upon the motif substrates of the *Homo sapiens* database. The most enriched extracted motifs for kinase interaction in both groups corresponded to proline directed MAPK kinases followed by Ca^2+^/calmodulin-dependent protein kinase II. Although in both cases, the number of peptide sequences that were aligned to the foreground dataset were higher in poly(I:C) treated cells compared to control. On the other hand, extracted motifs unique to cells treated with poly(I:C) corresponded mainly to Protein Kinases PKA, PKC, Casein Kinase II (CK2) and kinases P70 s6 and ZIP.

### Activation of Signaling Pathways

Several signaling pathways were activated in control and stimulated cells which were exclusively related to human pathways therefore phosphoproteins identified in rainbow trout were converted to human counter gene symbols. One of the pathways identified was the mitogen activated protein kinase (MAPK) pathway which was found to be activated in poly(I:C) stimulated cells (Supplementary Figure 2). In this study, Raf and MAP2K2 (MEK), the initiators of MAPK pathway, were phosphorylated. The p38 pathway was also found to be activated in poly(I:C) stimulated cells. A number of the intermediate proteins including MAP3K1, PAK2, MAPK14, MAPKAPK5 were found in the poly(I:C) group while phosphorylated MAP4K4 and JUN were detected in both groups. Another regulatory pathway related to cellular integrity was the actin cytoskeleton which was activated only in poly(I:C) stimulated cells (Supplementary Figure 3). The phosphoprotein associated genes of this pathway were fibronectin binding protein integrins itga4 and itga5, RRAS2 (homology of Ras), raf1a, PAK2 and ARHGEF6 all of which participate in the activation of regulation of actin cytoskeleton signaling.

## Discussion

The gills of fish are directly exposed to the external environment and can be a point of entry for many potentially pathogenic microorganisms. It has a complex architecture comprising several different types of cells making it difficult to study the role of each cell in host defense. The expression pattern of certain genes in gill homogenates taken during *in vivo* studies might be the cumulative effect of several cell types within the gill. As blood is continuously flowing through the gill, nucleated blood cells may also contribute to the observed expression of mRNA transcripts in the gills. Taken together it is therefore difficult to evaluate the specific contribution of gill epithelial cells to viral replication and innate immunity. Thus, epithelial RTgill-W1 cells have a great potential to unravel the specific role of gill epithelial cells in barrier function, viral replication and innate immunity.

Of particular note in the case of epithelial cells, *in vitro* studies often do not reflect an *in vivo* scenario as the epithelia is polarized *in vivo* but typically in conventional cell culture systems this beneficial cell orientations is lost. A more refined alternative can be deployed where epithelial cells are cultured in permeable inserts (transwells) that mimic the *in vivo* conditions and allow cell polarization (40). Polarized epithelial cells grown onto the transwells interact and uptake molecules from both apical and basal surfaces resembling an *in vivo* situation and likely allowing a faster and more coordinated response to stimuli. Therefore, the *in vitro* system used in this study is expected give a more accurate representation of the *in vivo* response of fish gills. Since the development of cell culture techniques, *in vitro* studies have become popular as this approach allows for consistent and reproducible experimentation. Cell culture also supports the 3Rs (replacement, refinement and reduction) agenda (https://www.nc3rs.org.uk/). However, in the case of epithelial cells *in vitro* studies do not reflect the *in vivo* situation as the epithelial cells typically develop polarization which is reduced or lost in conventional cell culture systems but can be retained in transwell culture. In the present study cellular integrity, measured by TEER, increased 3 h post-stimulation with poly(I:C). This observation was accompanied by a significant upregulation in the transcription, ~4-fold, of the tight junction regulatory gene ZO-1 at 24 h post-stimulation. TEER values returned to the baseline values at 5–7 days of post stimulation. A similar induction of TEER by poly(I:C) (10 μg/mL) has been reported in human epidermal keratinocytes (41) while Borkowski et al. (42) reported a dose-dependent increase of TEER along with claudin and occludin mRNA expression in human epidermal keratinocytes. Our results agree with these observations although modified expression of other tight junction genes was not found to be induced by poly(I:C) in RTgill-W1 cells. In contrast in a previous study in human polarized airway epithelial cells, poly(I:C) was shown to reduce TEER (43). In this study, post infection TEER in the SAV2 MOI-10 infected group decreased from as early as 30 min (time point at which phosphoproteins were analyzed) until 3 h post infection (hpi) and then significantly increased at 48 hpi. Therefore, further studies should elucidate regulation of the tight-junction response which appears in the transwell system to be very fast and transient.

In this study, the antiviral response of RTgill-W1 cells upon poly(I:C) stimulation and SAV-2 infection was investigated. The antiviral response in fish is initiated upon the sensing of viral PAMPs by the host's pattern recognition receptors (PRRs) including TLRs, RLRs, and NLRs (44). Both TLR3 and RLR recognize viral or synthetic dsRNA. While TLR3 recognizes dsRNA in the endosome, RLRs senses dsRNA in the cytoplasm and both trigger innate immune responses. TLR3 signaling is important for interferon-α/β mediated induction of ISGs and has been reported in several fish species including rainbow trout (45, 46) upon either poly(I:C) stimulation or viral infection (both dsRNA and ssRNA viruses). Zebrafish interferon has been shown to be induced by poly(I:C) in zebrafish liver cells (ZFL) (47). *In vivo* challenge with sole, *Solea senegalensis*, aquabirnavirus (solevirus) and poly(I:C) stimulation have been shown to induce Mx gene expression (48). In the present study, mRNA expression of TLR3, rtIFN2 and Mx2 was upregulated upon SAV-2 infection and poly(I:C) stimulation in RTgill-W1 cells. Moreover, the RLR family are expressed in the cytoplasm and regulate the production of interferon and ISGs, thus playing a major role in the antiviral response (44). TLR independent and RIG-I mediated antiviral responses have been reported in mammals upon viral infection (49–51). Poly(I:C) stimulation and viral infection have also been shown to upregulate RLR molecules and RLR family members in a wide range of fish species (52–54). In the present study three members of RLR molecules including RIG-I, MDA5, and LGP2b were upregulated upon poly(I:C) stimulation and +ssRNA viral infection in salmonid RTgill-W1 cells.

Integration of different molecules associated with specific signaling pathways is very important for immediate and coherent gene regulation through signal transduction against pathogens. Mechanisms associated to signal transduction encompass an array of strategies such as changes in gene expression, cellular localization, post-translational modifications and protein-protein interactions, all of which play critical roles allowing cells to respond with high specificity and efficiency (55). In this study, the expression of integration and response molecules associated with TLR3 and RLR signaling pathways including IPS1, TBK1, IRF3, Mx2, PKR, and viperin were found to be induced by SAV-2 and poly(I:C) in RTgill-W1 cells. From a cellular response perspective, the increased IPS-1-mediated interferon induction upon poly(I:C) stimulation was accompanied by the phosphorylation of 671 proteins, of which 390 were activated solely in response to the presence of poly(I:C). In this group, regulated phosphoproteins were mainly involved in signal transduction (MAPKs) and cytoskeleton regulation as shown by both GO and pathway analysis. According to motif analysis, kinases identified exclusively, or predominantly phosphorylated upon Poly(I:C) stimulation included MAPKs, Ca2+/calmodulin-dependent kinase (CaMK) and cAMP-dependent protein kinase (PKA), all reported to be activated in response to various viruses in human cells (56–60). Other kinases identified by motif analysis in the poly(I:C) stimulated group were Casein Kinase 2 (CK2) and p70 s6 kinase. CK2 has been reported to be activated at initial stages of viral infection (61). This highly conserved kinase has been shown to be involved in virus replication by phosphorylating viral antigens and nuclear phosphoproteins that modulate RNA replication (62). In fact, a wide number of virus families have been shown to rely on host CK2 for replication (61). On the other hand, p70 s6 kinase has been reported to be involved in virus RNA replication through the activation of PAK1, a mechanism that could be further studied by the use of specific inhibitors (63).

Interestingly, another mechanism unraveled by this untargeted approach was the activation of cytoskeleton reorganization. It is well-known that viruses reconfigure and reorganize cellular actin in order to initiate, sustain and spread the infection. However, the extent and degree of cytoskeletal reorganization depends on the viral agent/strategy (64). In viruses such as alphaherpesvirus, p21-activated kinase PAK2 is required for US3-mediated stress fiber disassembly which is fundamental for efficient virus spread in monolayers with an increase in the efficiency of intercellular virus spread (65). Actin stress fiber disassembly is generally associated with loss in focal adhesions and cell to cell contact (66). In relation to the activation of cytoskeleton reorganization it is worth noting the phosphorylation detected in integrin subunits α 4 and 5 (itga4 and itga5) in poly(I:C) stimulated cells. Integrins are a family of transmembrane receptor proteins that connect the cytoskeleton with components of the extracellular matrix and some of its members are known receptors or coreceptors for many viruses (67, 68). This effect could be one of the main drivers facilitating adhesion, cytoskeleton rearrangement, and increased intracellular signaling, all mechanisms found in virus-integrin interactions (69). While further work is now needed to validate the role of integrins in the transwell system results from the phosphoproteome analysis indicate that this could be an important mechanism for the interaction between viruses and the gill epithelia.

## Conclusion

The findings of the current study describe a rapid antiviral response in polarized trout gill epithelial cells. The results of gene expression studies confirm that RLR-mediated antiviral immunity is evolutionarily conserved throughout vertebrate history as a result of co-evolutionary history with host cells and viruses (44, 70, 71). Thus, antiviral immunity in the salmonid gill epithelium in response to viral infection and stimulation with viral particles is, of course, evolutionarily conserved from the earliest common ancestors. Furthermore, our results from the untargeted study of the phosphoproteome have identified kinases that play a key role upon viral infection such as MAPKs, Ca2+/calmodulin-dependent kinase (CaMK) and cAMP-dependent protein kinase (PKA), all reported to be activated in response to various viruses in mammals. Results also highlight activation of the cytoskeletal organization likely mediated by members of the integrin family. While further work is now necessary to validate these results our data indicates that polarized RTgill-W1 cell cultures provide an excellent model to further our understanding of host-pathogen interactions at the fish gill surface.

## Data Availability Statement

The datasets presented in this study can be found in online repositories. The names of the repository/repositories and accession number(s) can be found in the article/Supplementary Material.

## Author Contributions

SM conducted the experiment, analyzed the data, and prepared the first draft. MW, AA, and SM designed the experiment and supervised the study. EC performed the LC/MSMS and interpreted the data. BM developed the phosphoproteome experiment and analyzed the data. All authors contributed in writing the manuscript and approved the final version.

## Conflict of Interest

The authors declare that the research was conducted in the absence of any commercial or financial relationships that could be construed as a potential conflict of interest.
