# Supplementary material for: Polarized Trout Epithelial Cells Regulate Transepithelial Electrical Resistance, Gene Expression, and the Phosphoproteome in Response to Viral Infection
Source: Front Immunol. 2020 Aug 14;11:1809. doi: 10.3389/fimmu.2020.01809 (PMC7456818; doi:10.3389/fimmu.2020.01809)
Supplement: Supplementary file 2 [file Data_Sheet_2.docx]

Supplementary Material

# Supplementary Data

**Supplementary data file 1 (attached as an excel file).** List of phosphoproteins with relevant information including Uniprot and NCBI accession number, molecular weight (Kda), functions and functional groups, sub-cellular localization, mean abundance of each protein of each group and p-value of the proteins detected in both the groups.

# Supplementary Figures and Tables

## Supplementary Figures

**
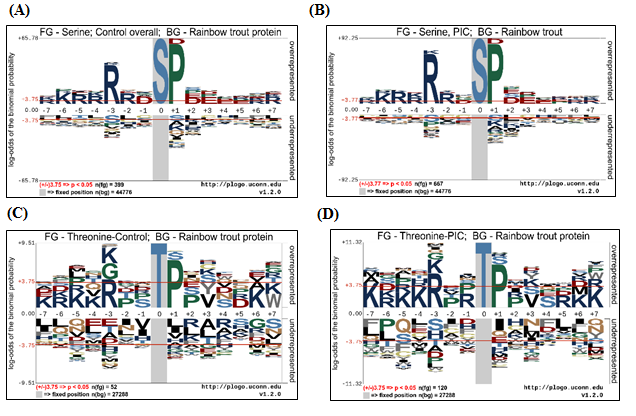
**

**Supplementary figure 1.** Overall pLogos for motifs of control and poly(I:C) treated cells illustrated by serine (S) top two (A-B) and threonine (T) bottom two (C-D). pLogos were derived from phosphorylation sites in rainbow trout (*Oncorhynchus* *mykiss*) phosphopeptides. In each pLogo, residue heights are proportional to their log binomial probabilities in the context of the rainbow trout protein background with residues above the x-axis indicating overrepresentation and residues below the x-axis indicating underrepresentation (p<0.05). The central residue in each pLogo is fixed and denotes the phosphorylation site. The n(fg) and n(bg) values at the bottom of each pLogo indicate the number of aligned foreground and background sequences respectively.


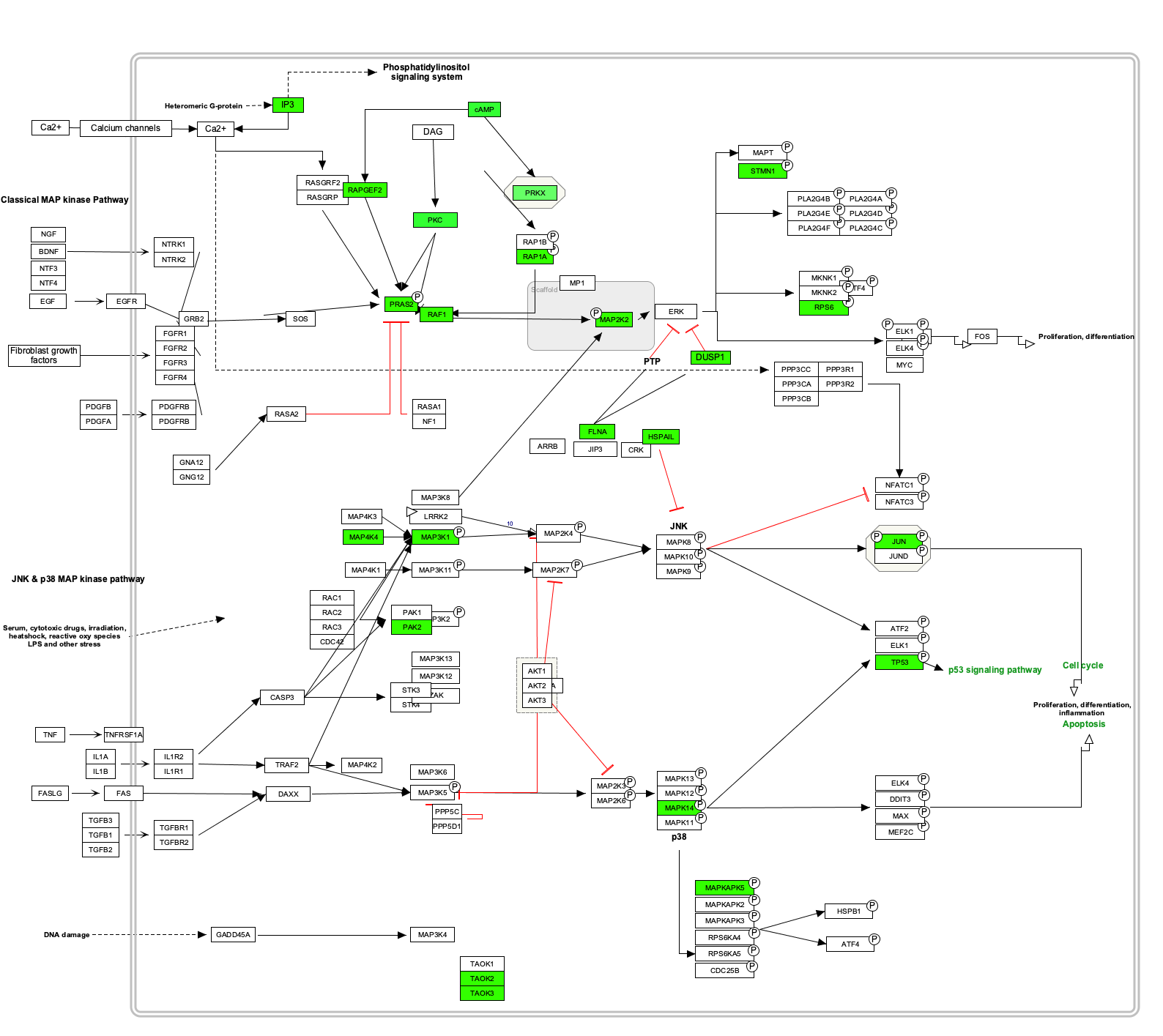


Supplementary figure 2. MAPK signaling pathway. The pathway was adopted from the KEGG pathway database for Homo sapiens (pathway id hsa04010; https://www.genome.jp/kegg-bin/show_pathway?org_name=hsa&mapno=04010&mapscale=&show_description=hide). Phosphoproteins in green filled are associated with the phosphorylated proteins identified only in poly(I:C) stimulated RTgill-W1 cells.


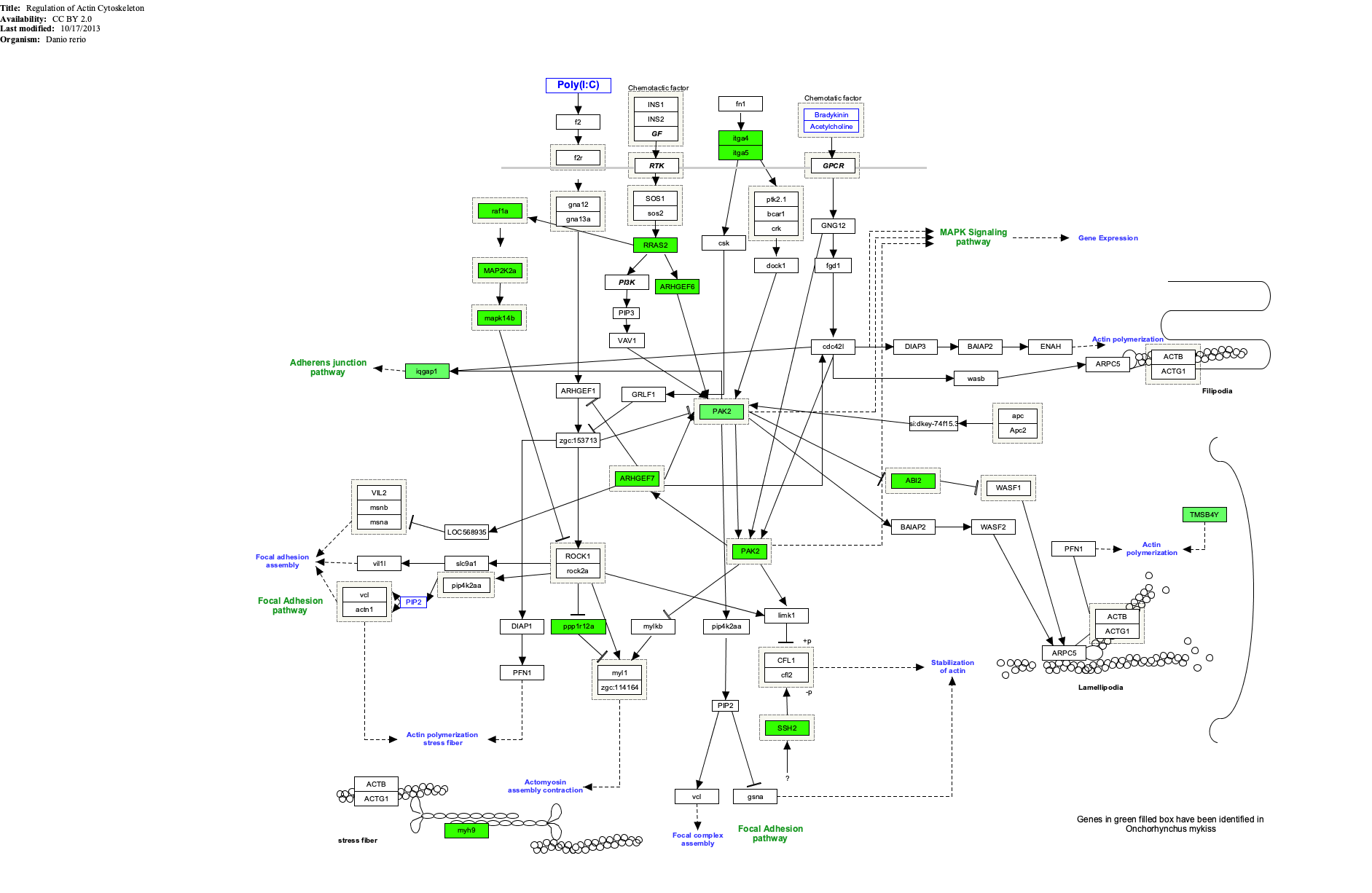


Supplementary figure 3. ﻿Regulation of actin cytoskeleton pathway. The pathway was adopted from the KEGG pathway database for Homo sapiens (pathway id hsa04810; https://www.genome.jp/kegg-bin/show_pathway?org_name=hsa&mapno=04810&mapscale=&show_description=hide). Phosphoproteins in green filled are associated with the phosphorylated proteins identified only in poly(I:C) stimulated RTgill-W1 cells.

## Supplementary Tables

**Supplementary Table 1.** List of motifs and kinases identified from the phosphorylation sites in RTgill-W1 cells in the study. Number in the parenthesis represents percentage probability. ^a^Foreground data set is the sequences within which a motif is searched, and the number is the total peptide sequences that are aligned to the foreground dataset. ^b^Background data set is the proteome of specific organism from where the probability of specific residue of foreground data is determined. The motifs were derived from the pLogos of control and poly(I:C) treated cells. pLogos were derived from phosphorylation sites in rainbow trout (*Oncorhynchus* *mykiss*) phosphopeptides.

| Motif | Kinases | Foreground matching | | Background matching | | References |
| --- | --- | --- | --- | --- | --- | --- |
|  |  | Control | PIC | Control | PIC |  |
| .......sP...... | Proline directed MAPK | 156 | 242 | 3910 | 3910 | (Schwartz and Gygi, 2005) |
| ....R..s....... | CaMK II | 121 | 210 | 3455 | 3455 | (Pearson and Kemp, 1991) |
| .K.....s....... |  | 63 | 95 | 2721 | 2721 |  |
| .....R.s....... | PKB | 61 | 96 | 2653 | 2653 | (Pearson and Kemp, 1991) |
| ..R....s....... | Casein II | 64 | 111 | 3102 | 3102 | (Pinna and Ruzzene, 1996) |
| ...R...s....... | cGMP dependent protein kinase | 58 | 85 | 2900 | 2900 | (Pearson and Kemp, 1991) |
| .......sD...... | CKII | 51 | 68 | 2464 | 2372 | (Villen et al., 2007) |
| .......s......R |  | 58 | 80 | 3034 | 3034 |  |
| R......s....... |  | 48 | 72 | 2989 | 2989 |  |
| .......s..E.... | CKII | 63 | 97 | 3476 | 3476 | (Villen et al., 2007) |
| .......s.D..... | CaMK II | 49 | 77 | 2500 | 2500 | (Schwartz and Gygi, 2005) |
| .......s.....R. |  | 46 | 68 | 2835 | 2835 |  |
| .......s.E..... | G-CK | 49 | 79 | 3128 | 3128 | (Schwartz and Gygi, 2005) |
| .....R.sP...... |  | 29 | 38 | 270 | 270 |  |
| .......sP.....R |  | 30 | 36 | 326 | 326 |  |
| ....R..sP...... |  | 35 | 51 | 430 | 430 |  |
| ....R..s..P.... |  | 26 | 37 | 35 | 351 |  |
| .K....Ds....... |  | 17 |  | 180 |  |  |
| .KR....s....... |  | 16 | 22 | 242 | 242 |  |
| ....RR.s....... | PKA kinase | 22 | 32 | 320 | 320 | (Pearson and Kemp, 1991) |
| ..R....s..E.... |  | 19 | 21 | 231 | 231 |  |
| ..R...Ds....... |  | 12 | 14 | 152 | 152 |  |
| ..R....sD...... |  | 14 |  | 182 |  |  |
| ......DsD...... | CK-II like | 18 | 23 | 256 | 256 | (Schwartz and Gygi, 2005) |
| ......Ds..E.... |  | 18 | 22 | 266 | 266 |  |
| ....R..s......R |  | 21 |  | 403 |  |  |
| R......sP...... |  | 19 | 27 | 326 | 326 |  |
| .......s.D..E.. | CK | 15 |  | 238 |  | (Villen et al., 2007) |
| ....R..sD...... |  | 11 | 20 | 147 | 147 |  |
| .......sD.E.... | Casein II | 21 | 33 | 297 | 297 | (Schwartz and Gygi, 2005) |
| ....R..s.....R. |  | 16 |  | 351 |  |  |
| .K.....s.E..... |  | 12 |  | 230 |  |  |
| ......Ds.E..... |  | 13 | 19 | 221 | 221 |  |
| .......sP..E... |  | 14 | 26 | 232 | 232 |  |
| ....R..s...E... |  | 18 | 31 | 272 | 272 |  |
| .K.....s...E... |  | 14 |  | 208 |  |  |
| ..R....s...E... |  | 12 |  | 230 |  |  |
| ......Ds....... |  |  | 65 |  | 2464 |  |
| .......sDE..... | Casein II |  | 16 |  | 213 | (Schwartz and Gygi, 2005) |
| ...R...sP...... |  |  | 22 |  | 294 |  |
| ......Ds....... | CK |  | 65 |  | 2464 | (Villen et al., 2007) |
| .......s.....R. |  | 46 | 68 | 2835 | 2835 |  |
| ...K...s....... | PKA kinase substrate |  | 77 |  | 2864 | (Pearson and Kemp, 1991) |
| .......s.S..... |  |  | 141 |  | 6531 |  |
| .......s...E... | CKII |  | 82 |  | 3338 | (Villen et al., 2007) |
| ....K..s....... | PKA kinase |  | 76 |  | 2824 | (Pearson and Kemp, 1991) |
| .......s..P.... |  |  | 78 |  | 3403 |  |
| .......s.P..... |  |  | 76 |  | 3278 |  |
| .......sP....R. |  |  | 39 |  | 312 |  |
| ....RT.s....... |  |  | 24 |  | 168 |  |
| ..R.R..s....... | p70 S6 kinase |  | 35 |  | 535 | (Leighton *et al.*, 1995) |
| ..R....sP...... |  |  | 31 |  | 349 |  |
| K......sP...... |  |  | 24 |  | 225 |  |
| K.....Ds....... |  |  | 18 |  | 180 |  |
| K......s...E... |  |  | 20 |  | 208 |  |
| .......sED..... |  |  | 17 |  | 206 |  |
| .......s.D.E... | CKII |  | 21 |  | 238 | (Villen *et al.*, 2007) |
| ....R..s..E.... |  |  | 31 |  | 240 |  |
| ...RR..s....... | ZIP kinase |  | 29 |  | 393 | (Burch *et al.*, 2004) |
| ...KR..s....... | PKA/PKC kinase |  | 33 |  | 286 | (Pearson and Kemp, 1991) |
| ...K...sP...... |  |  | 23 |  | 217 |  |
| ....K..s......R |  |  | 27 |  | 403 |  |
| ....R..s.S..... |  |  | 56 |  | 746 |  |
| ..R....s.S..... |  |  | 29 |  | 582 |  |
| .......sPS..... |  |  | 50 |  | 596 |  |
| .......s.SP.... |  |  | 35 |  | 690 |  |
| ....R..s.SP.... |  |  | 20 |  | 150 |  |
| .......sPSP.... |  |  | 16 |  | 117 |  |
| ....R..s.E..... |  |  | 21 |  | 258 |  |
| .......sDEE.... | CKII |  | 12 |  | 58 | (Villen *et al.*, 2007) |
| .......s.EE.... |  |  | 18 |  | 466 |  |
| .......s...E..R |  |  | 15 |  | 207 |  |
| .....DDs....... |  |  | 14 |  | 156 |  |
| ....K..sP...... |  |  | 17 |  | 244 |  |
| R...R..s....... |  |  | 24 |  | 409 |  |
| ..K....sD...... |  |  | 18 |  | 171 |  |
| .......s..R..R. |  |  | 21 |  | 351 |  |
| .......sPP..... |  |  | 35 |  | 346 |  |
| ......Gs....... |  |  | 72 |  | 3050 |  |
| ......GsP...... |  |  | 20 |  | 247 |  |
| ....R.Gs....... |  |  | 18 |  | 246 |  |
| .......tP...... | Proline directed MAPK | 18 | 30 | 2252 | 2252 | (Schwartz and Gygi, 2005) |
| ...R...t....... | cGMP dependent protein kinase | 11 | 21 | 1427 | 1427 | (Pearson and Kemp, 1991) |
| K......t....... |  |  | 21 |  | 1845 |  |
| ...R...tP...... |  |  | 10 |  | 115 |  |

**References**

Burch, L.R., Scott, M., Pohler, E., Meek, D., Hupp, T. (2004). Phage-peptide Display Identifies the Interferon-responsive, Death-activated Protein Kinase Family as a Novel Modifier of MDM2 and p21WAF1. J. Mol. Biol. 337, 115–128. https://doi.org/10.1016/j.jmb.2003.10.081

Leighton, I.A., Dalby, K.N., Barry Caudwell, F., Cohen, P.T.W., Cohen, P. (1995). Comparison of the specificities of p70 S6 kinase and MAPKAP kinase-1 identifies a relatively specific substrate for p70 S6 kinase: the N-terminal kinase domain of MAPKAP kinase-1 is essential for peptide phosphorylation. FEBS Lett. 375, 289–293. https://doi.org/10.1016/0014-5793(95)01170-J

Pearson, R.B., Kemp, B.E. (1991). Protein Kinase Phosphorylation Site Sequences and Consensus Specificity Motifs: Tabulations. Methods Enzymol. 200, 62–81.

Pinna, L.A., Ruzzene, M. (1996). How do protein kinases recognize their substrates? Biochim. Biophys. Acta - Mol. Cell Res. 1314, 191–255. https://doi.org/10.1016/S0167-4889(96)00083-3

Schwartz, D., Gygi, S.P. (2005). An iterative statistical approach to the identification of protein phosphorylation motifs from large-scale data sets. Nat. Biotechnol. 23, 1391–1398. https://doi.org/10.1038/nbt1146

Villen, J., Beausoleil, S.A., Gerber, S.A., Gygi, S.P. (2007). Large-scale phosphorylation analysis of mouse liver. Proc. Natl. Acad. Sci. 104, 1488–1493. https://doi.org/10.1073/pnas.0609836104
